# WDR73 Depletion Destabilizes PIP4K2C Activity and Impairs Focal Adhesion Formation in Galloway–Mowat Syndrome

**DOI:** 10.3390/biology11101397

**Published:** 2022-09-25

**Authors:** Hongyan Li, Fang Liu, Hanzhe Kuang, Hua Teng, Siyi Chen, Sijing Zeng, Qimin Zhou, Zhaokai Li, Desheng Liang, Zhuo Li, Lingqian Wu

**Affiliations:** Center for Medical Genetics, Hunan Key Laboratory of Medical Genetics & Hunan Key Laboratory of Animal Models for Human Disease, School of Life Sciences, Central South University, Changsha 410000, China

**Keywords:** WDR73, PIP4K2C, PIP2, focal adhesion

## Abstract

**Simple Summary:**

Galloway–Mowat syndrome is a rare genetic disease, classically characterized by a combination of various neurological symptoms and nephrotic syndrome. WDR73 is the pathogenic gene responsible for Galloway–Mowat syndrome. However, the pathological and molecular mechanisms of Galloway–Mowat syndrome, especially nephrotic syndrome caused by WDR73 deficiency, remains unknown. In this study, we knocked out the WDR73 in human embryonic kidney 293 cells to observe the morphological characteristics of the cells and elucidate the functions of WDR73. Additionally, we used a combination of proteomics, transcriptomics, and biochemical assays to identify the regulated targets of WDR73. We aimed to discover directly interacting molecules and the regulatory pathway of WDR73 and to illustrate the molecular mechanism between the WDR73 pathway and nephrotic disease in Galloway–Mowat syndrome. From the molecular mechanism we found in vitro, we draw a hypothesis that the damage to focal adhesion of podocytes caused by WDR73 defect is the key issue of kidney dysfunction. Finally, we verified the hypothesis in a podocyte-specific conditional knockout Wdr73 mouse model.

**Abstract:**

(1) Background: Galloway–Mowat syndrome (GAMOS) is a rare genetic disease, classically characterized by a combination of various neurological symptoms and nephrotic syndrome. WDR73 is the pathogenic gene responsible for GAMOS1. However, the pathological and molecular mechanisms of GAMOS1, especially nephrotic syndrome caused by WDR73 deficiency, remain unknown. (2) Methods and Results: In this study, we first observed remarkable cellular morphological changes including impaired cell adhesion, decreased pseudopodia, and G2/M phase arrest in WDR73 knockout (KO) HEK 293 cells. The differentially expressed genes in WDR73 KO cells were enriched in the focal adhesion (FA) pathway. Additionally, PIP4K2C, a phospholipid kinase also involved in the FA pathway, was subsequently validated to interact with WDR73 via protein microarray and GST pulldown. WDR73 regulates PIP4K2C protein stability through the autophagy–lysosomal pathway. The stability of PIP4K2C was significantly disrupted by WDR73 KO, leading to a remarkable reduction in PIP2 and thus weakening the FA formation. In addition, we found that podocyte-specific conditional knockout (Wdr73 CKO) mice showed high levels of albuminuria and podocyte foot process injury in the ADR-induced model. FA formation was impaired in primary podocytes derived from Wdr73 CKO mice. (3) Conclusions: Since FA has been well known for its critical roles in maintaining podocyte structures and function, our study indicated that nephrotic syndrome in GAMOS1 is associated with disruption of FA caused by WDR73 deficiency.

## 1. Introduction

Galloway–Mowat syndrome (GAMOS; OMIM 251300) is a rare recessive genetic disease characterized by neurodevelopmental defects and progressive renal glomerulopathy. Neurological involvement of GAMOS contains microcephaly, developmental delay, intellectual disability, and other variable neural symptoms [1]. Renal manifestations range from isolated proteinuria to early nephrotic syndrome (NS), which may rapidly progress to end-stage renal disease. The prognosis of GAMOS is poor, and the majority of affected children died before six years of age due to therapy-resistant renal failure [2,3].

To date, mutations in 11 different genes, such as WDR73, KEOPS complex genes, YRDC, and NUP107, have been reported to cause GAMOS [4,5,6,7]. Although WDR73 is the first gene to be identified in GAMOS, the molecular mechanisms underlying the pathophysiology of GAMOS upon WDR73 function remain obscure. WDR73 encodes a WD40-repeat-containing protein that can be expressed in a variety of cells. In the fetal kidney, WDR73 exists in immature podocytes from the S-shaped body stage to the capillary-loop stage. In mature glomeruli, WDR73 has a punctate distribution at the periphery of the glomerular tuft and is present in the cell bodies of mature podocytes. WDR73 is localized in the cytoplasm during interphase and accumulates at spindle poles and microtubule asters during mitosis; thereby, it may play a role in cellular architecture and cell cycle [4]. It is reported that WDR73 could interact with α- and β-tubulin in fibroblasts [8]. Loss of WDR73 also has been demonstrated to disrupt the integrator complex, perturbing the transcription of cell cycle regulatory proteins [9]. Despite these observations providing some insights into the function of WDR73, the regulatory pathway of WDR73, the precise contributions of WDR73 to cell physiological function, and the mechanisms underlying GAMOS, especially nephrotic syndrome caused by WDR73 deficiency, are poorly understood.

In this study, we knocked out the WDR73 in human embryonic kidney (HEK) 293 cells to observe the morphological characteristics of the cells and elucidate the functions of WDR73. Additionally, we used a combination of proteomics, transcriptomics, and biochemical assays to identify the regulated targets of WDR73. We aimed to discover directly interacting molecules and the regulatory pathway of WDR73 and to illustrate the molecular mechanism between the WDR73 pathway and nephrotic disease in Galloway–Mowat syndrome. From the molecular mechanism we found in vitro, we draw a hypothesis that the damage to focal adhesion of podocytes caused by WDR73 defect is the key issue of kidney dysfunction. Finally, we verified the hypothesis in a Wdr73 CKO mouse model.

## 2. Materials and Methods

### 2.1. Cell Culture and Reagents

HEK 293 cells were cultured in DMEM medium supplemented with 10% FBS and 1% penicillin–streptomycin (Thermo Fisher Scientific, Waltham, MA, USA) at 37 °C under 5% CO2. Transfections were performed using Lipofectamine 3000 (Thermo Fisher Scientific, Waltham, MA, USA), following the manufacturer’s instructions. The following reagents were used in this study: cycloheximide (CHX), MG132, and chloroquine (Selleck, Houston, TX, USA).

### 2.2. WDR73-Knockout (KO) and Stable Cell Lines

HEK293-WDR73 KO cells were generated using the CRISPR/Cas9 system. Briefly, plasmids carrying sgRNA targeting the WDR73 region encompassing exon 6 were introduced into HEK293 cells using Lipofectamine 3000. Transfected HEK293 colonies were selected using puromycin (Solarbio, Beijing, China). The puromycin-selected colonies were seeded into 96-well plates (1 cell per well) and expanded. Furthermore, WDR73 with frameshift deletions in the six exons was confirmed by Sanger sequencing. Western blotting was used to examine WDR73 protein expression in each clone. WDR73 KO cells were transfected with plasmids carrying WT WDR73 using Lipofectamine 3000 and selected using hygromycin. Cell lines carrying the stably integrated plasmid were expanded from a single colony. The colony with an approximate protein expression level was selected.

### 2.3. Plasmids and Antibodies

Plasmids were constructed as follows: Plasmids expressing WT WDR73 were generated by cloning human WDR73 into pcDNA3.1-Hygro vector. For co-immunoprecipitation, a plasmid expressing 3×HA-WDR73 was generated by cloning human WDR73 into an in-house modified version of the pcDNA3.1(+) -3×HA vector. PCMV-GFP-hEPG5 was kindly provided by Professor Hong Zhang (Institute of Biophysics, Chinese Academy of Sciences, China). Plasmids expressing GFP were generated by cloning GFP into a PCMA vector. For the GST pulldown assay, PIP4K2C was PCR-amplified and cloned into the pGEX-6P-1 vector to produce GST-tag fused recombinant proteins, whereas WDR73 was PCR-amplified and cloned into the pET28A vector to produce His-tag fused recombinant proteins. Plasmids for WDR73 KO were constructed by inserting sgRNA sequences into the pSpCas9(BB)-2A-Puro (PX459) V2.0 plasmid. The sgRNA sequences specifically targeting WDR73 were as follows: F, CACCGACTTCGGAGCCTCGCCCCA; R, AAACTGGGGCGAGGCTCCGAAGTC. The antibodies used in this study are shown in Table S4.

### 2.4. Cell Proliferation Assay

The proliferative ability of the cells was measured using the CCK-8 assay (Vazyme, Nanjing, China) according to the manufacturer’s instructions. Briefly, cells were plated into a 96-well plate at a density of 2.0 × 10^3^ cells/well and incubated at 37 °C for 0, 24, 48, 72, and 96 h. Twenty microliters of CCK-8 reagent was added to each well, and the cells were cultured for 2 h. All experiments were performed in triplicates. Absorbance was measured at 450 nm using a microplate reader (ELx800, BioTek, Winooski, VT, USA).

### 2.5. Cell Cycle and Apoptosis Assays

The cells were harvested after 72 h, and the cell suspension was then digested. Next, the cells were fixed using ethanol (75%) for 4 h at 4 °C, and the supernatant was subsequently discarded, followed by incubation with an RNA enzyme containing iodide (PI, Sigma-Aldrich, St. Louise, MO, USA). After the cells were washed three times with PBS, the cell cycle was detected using a Cytek Dxp Athena flow cytometer (Cytek, Biosciences, San Diego, CA, USA), and data analysis was conducted using the Modfit LT software. For the determination of apoptosis, the cells were stained with FITC-conjugated annexin V and PI according to the manufacturer’s instructions (Vazyme, Nanjing, China). Data were collected and analyzed using a Cytek Dxp Athena flow cytometer, and data analysis was performed using the FlowJo software 10.4 (BD, Ashland, USA). All experiments were performed in triplicates.

### 2.6. Cell Adhesion and Spreading Assay

For all cell lines, 2.0 × 10^3^ cells/well were seeded onto 96-well plates, and four regions per well were imaged every 2 h over a period of 48 h using the Incucyte Zoom (Essen BioScience, Ann Arbor, MI, USA). The cell areas were measured using the Incucyte Zoom software package to obtain quantitative data on the extent of cell spreading.

### 2.7. Immunofluorescence

The coverslip-grown cells were fixed in 4% paraformaldehyde for 20 min at room temperature or in ice-cold MeOH for 5 min. The coverslips were permeabilized in 0.5% Triton X-100/PBS for 15 min and then blocked in 5% bovine serum albumin/PBS for 1 h at room temperature. Primary antibodies were applied in a dilution according to the instructions on staining buffer overnight at 4 °C. Secondary antibodies (Alexa Fluor secondary 488; cy3, Jackson, West Grove, PA, USA) were applied in a 1:200 dilution in staining buffer for 1 h at 37 °C in the dark. The nuclei were stained with DAPI. Actin filaments were labeled with FS488 phalloidin (Solarbio, Beijing, China). All images were captured using a confocal laser scanning microscope (Zeiss LSM 880 with airyscan, Zeiss, Berlin, Germany). The primary antibodies used were described previously.

### 2.8. Human Proteome Microarray Assays

The recombinant His-WDR73 fusion protein was purified by MerryBio Co., Ltd. (Huai’an, China). After quantification and qualification, the recombinant His-WDR73 fusion proteins were labeled with Cy3 (CyDye Protein LabellingCY3 MONO 5-PACK, GE) and used to probe the Arrayit HuProt v4.0 20 K Human Proteome Microarrays (CDI Laboratories, Baltimore, MD, USA). Briefly, Cy3-tagged His-WDR73 was incubated on the microarray overnight at 4 °C and washed. Finally, the microarray was scanned using a microarray scanner (CapitalBio, Beijing, China). Data were analyzed to generate a candidate list of WDR73-binding proteins; the signal-to-noise ratio (SNR) was defined as the ratio of the median foreground value to the median background value. A total of 336 candidates were selected based on a Z-score > 3 (Z-score = (SNR-mean)/SD). Gene Ontology (GO) enrichment analysis and Kyoto Encyclopedia of Genes and Genomes (KEGG) pathway analysis were conducted to reveal the unique biological significance and key pathways associated with WDR73 in the candidate list of WDR73-binding proteins (criteria: *p*-value < 0.05, significantly enriched).

### 2.9. Western Blotting

Cells were harvested and lysed using RIPA lysis buffer (Beyotime, Haimen, China) containing 1 mM PMSF and 1× protease inhibitor cocktail (Sigma-Aldrich, St. Louise, MO, USA). Cell lysates were separated using SDS-PAGE and transferred to a PVDF membrane (Merck Millipore, Burlington, MA, USA). The membranes were blocked in Tris-buffered saline containing 0.01% Tween20 and 5% non-fat milk for 1 h and then incubated with specific primary antibodies. Following incubation with horseradish peroxidase (HRP)-linked secondary antibody (Jackson, West Grove, PA, USA) at room temperature for 1 h, detection was performed using an Immobilon Western Chemiluminescent HRP substrate kit (Thermo Fisher Scientific, Waltham, MA, USA) according to the manufacturer’s instructions. The primary antibodies used were as previously described.

### 2.10. GST Pulldown Assays

The plasmids encoding GST, GST-PIP4K2C, and His-WDR73 were transfected into *E. coli*. The fusion proteins were prepared as previously described. Approximately 100 µg of GST and GST-PIP4K2C fusion protein was immobilized in 50 µL of Mag-Beads GST Fusion Protein Purification (Sangon Biotech, Shanghai, China) and equilibrated before being incubated together at 4 °C for 60 min with a gentle rocking motion. Approximately 100 µg of His-WDR73 fusion protein was added to immobilized GST-PIP4K2C and GST after three washes with PBST. The two fusion proteins were incubated overnight at 4 °C with gentle agitation. The bound proteins were washed five times with PBS, boiled with loading buffer for 5 min, and analyzed by Western blotting, as described previously.

### 2.11. Co-Immunoprecipitation (Co-IP)

HEK293 cells were transfected with plasmids expressing 3HA-WDR73, GFP-Epg5, 3HA-WDR73, and GFP using Lipofectamine 3000 (Invitrogen Waltham, MA, USA). Cells were lysed using immunoprecipitation buffer (20 mM Tris (pH7.5), 150 mM NaCl, 1% Triton X-100, 1 mM PMSF, and 1× protease inhibitor cocktail (Sigma-Aldrich St. Louise, MO, USA) 48 h after transfection. For Co-IP, approximately 1000 μg protein was incubated at 4 °C overnight with 30 μL indicated monoclonal Anti-HA magnetic beads (Bimake, Houston, TX, USA). The precipitates were washed three times with immunoprecipitation buffer, boiled with loading buffer for 5 min, and analyzed by Western blotting, as described previously.

### 2.12. PI(4,5)P2 ELISA Assay

PIP2 was quantified using a PI(4,5)P2 mass ELISA kit (echelon, K-4500, Salt Lake City, UT, USA). Briefly, lipids were extracted from WDR73 KO and WT cells according to the manufacturer’s instructions. The PIP2 levels were measured according to the manufacturer’s instructions. The absorbance was measured at 450 nm using a microplate reader (ELx800, BioTek, Winooski, VT, USA). Finally, the quantity of PIP2 was calculated using a standard curve.

### 2.13. RNA Sequencing

Total RNA was extracted from WDR73 KO and WT cells using TRIzol reagent (Invitrogen, Waltham, MA, USA). After RNA quantification and qualification, library preparation and transcriptome sequencing were performed using Novogene Bioinformatics Technology (Beijing, China). Differential expression analysis was performed using the DESeq2 R package. Finally, enrichment analysis of differentially expressed genes (DEGs), GO enrichment analysis, and KEGG pathway analysis were conducted using clusterProfiler (Bioconductor, Boston, MA, USA) to reveal the unique biological significance and key pathways associated with WDR73 in the DEGs (criteria: *p*-value < 0.05, significantly enriched). The Disease Ontology (DO) database describes the function of human genes and diseases, and we used clusterProfiler software to test the statistical enrichment of DEGs in the DO pathway (criteria: *p* < 0.05, significantly enriched).

### 2.14. RNA Isolation and Quantitative Reverse Transcription (qRT-PCR)

Total RNA was extracted from cells using TRIzol reagent (Invitrogen, Waltham, MA, USA), and mRNA was reverse transcribed using a RevertAid First Strand cDNA Synthesis Kit (Thermo Fisher Scientific, Waltham, MA, USA) according to the manufacturer’s instructions. The real-time PCR assay was conducted using 2× SYBR Green Mix (Thermo Fisher Scientific, Waltham, MA, USA) on an ASA-9600 qRT-PCR System (Lanzhou Baiyun Gene Technology Co., Ltd, Lanzhou, China). Primers used for real-time PCR are listed in Tables S2, S3 and S5. Relative expression was calculated using the 2^−ΔΔCt^ method.

### 2.15. Mice

Mice were housed in a specific-pathogen-free facility and kept in a 12 h day/night cycle with free access to chow and water. Genotyping and breeding of animals were performed according to standard procedures. Wdr73 general KO mice were generated by Shanghai Biomodel Organisms Center using CRISPR/Cas9. A guide RNA targeting the KO exon 6–exon 8 of the Wdr73 (ENSMUST00000026816.14) gene was designed. The guide RNA1 and guide RNA2 sequences are shown in Table S5. Male Wdr73 frameshift heterozygous mice and female Wdr73 frameshift heterozygous mice were mated to obtain homozygous mice. For genotype identification, the Wdr73 frameshift mutation was identified by PCR amplification using the primers PI, PII, PIII, and PIV; (Table S5). All mouse lines were maintained in the C57BL/6J background by regular backcrossing to the C57BL/6J line. Study protocols complied with all relevant ethical regulations and were approved by the IRB of Central South University (IRB: 2019-2-17).

Wdr73 flox mice were generated by GemPharmatech Co., Ltd. (Nanjing, China), using CRISPR/Cas9 on a C57BL/6 background. These mice carry a cassette with LoxP sites flanking a region containing 154 bp coding sequence of Wdr73 exon 4–exon 5, the knocking out of which will result in disruption of protein function. For the generation of a podocyte-specific Wdr73 knockout mouse model, Wdr73flox/flox mice were crossed with Npsh2-Cre mice on a C57BL/6J background (GemPharmatech Co., Ltd, Nanjing, China). In the nephropathy experiments, male and female mice (aged 5–8 weeks), were treated with a single retroorbital injection of ADR (doxorubicin HCl; Macklin, shanghai, China) at the dose of 20 mg/kg for Nphs2-Cre, Wdr73flox/flox (Wdr73 CKO), and Wdr73flox/flox (WT) mice. For genotype identification, the Wdr73 flox was identified by PCR amplification using the primers Wdr73-5F/R and Wdr73-3F/R; Cre recombinase was identified by PCR amplification using the primers Nphs2-cre F/R (Table S5). General PCR amplification was performed to verify Wdr73 gene knockout efficiency using the primers Wdr73-5F and Wdr73-3R (Table S5).

The age of animals used for the respective experiments is stated in the figures and/or figure legends (male and female animals showed similar phenotypes and were combined for the analysis).

### 2.16. Measurement of Urinary Albumin and Creatinine

Albumin and creatinine levels were quantified by measuring spot urine from Wdr73 CKO and WT mice at defined time points. Proteinuria was expressed as albumin–creatinine ratio. Assessment of urinary albumin was performed using a mouse-specific, albumin fluorescence-based kit (Fankewei, Shanghai, China). Measurement of creatinine was performed using an enzymatic creatinine kit (Fankewei, Shanghai, China).

### 2.17. Urine Analysis for Albumin by SDS–PAGE

First, 16 μL of collected spot urine was mixed with 4 μL 4 × LDS Sample Buffer (NP0007, Invitrogen, Waltham, MA, USA); the sample was heated at 70 °C for 10 min for optimal results and separated by SDS–PAGE. Gels were stained with Coomassie for 1 h and destained using standard methods.

### 2.18. Renal Ultrastructural Analysis

Fresh kidneys underwent primary fixation with 2% glutaraldehyde in PBS. They were then postfixed in 1% osmium tetroxide for 1 h and dehydrated in 50%, 70%, 90%, 95%, or 100% ethanol and propylene oxide for 10 min each. Samples were further infiltrated with an epoxy resin mixture. Ultrathin sections were collected on copper grids, and sections were stained using 10% uranyl acetate in 50% methanol and modified Sato lead stain. A HITACHI HT7700 electron microscope was used for picture acquisition (Lab of Biomedical Electronic Microscopy Higher Research Center, Central South University, Changsha, China).

### 2.19. Histology Staining of Kidney Sections

Fresh kidney immersion was fixed in 4% PFA in phosphate-buffered saline (PBS) for 24 h and subsequently dehydrated in 20% or 30% sucrose solution for 24 h each at 4 °C. Kidney cryosections of O.C.T.-embedded (20 μm thick) (Sakura TissueTek #4583, Sakura Finetek, Alphen aan den Rijn, the Netherlands) and kidney sections (3–4 μm thick) of paraffin-embedded (FFPE) tissue were generated using standard methods. FFPE sections were deparaffinized and rehydrated and then underwent heat-induced antigen treatment.

### 2.20. Mice Glomerular Isolation and Podocyte Culture

Glomeruli were isolated from male or female mice aged between 5 and 8 weeks old using previously described methods [10]. Mice are anesthetized with Avertin (250 mg/kg body weight) and perfused via the left heart ventricle with 20–40 mL PBS. Isolated kidneys were minced in ice PBS and digested with collagenase type II (2 mg/mL; C5138, Sigma-Aldrich St. Louise, MO, USA) at 37 °C for 30 min, and glomeruli were sequentially sieved with a 100 μm and 70 μm cell strainer in order and washed with PBS. The isolated glomeruli were seeded on collagen I (C8062, Solarbio, Beijing, China)-coated plates. The method yields primary podocytes with 90% purity, confirmed via staining with the podocyte-specific marker nephrin on day 24 after isolation. Primary mouse podocytes were cultured in RPMI medium (Gibco, Waltham, MA, USA) supplemented with 10% FBS and 1% penicillin–streptomycin (Thermo Fisher Scientific Waltham, MA, USA) at 37 °C under 5% CO2, and they were fed with fresh medium every 2–3 days.

### 2.21. EdU Assay of Primary Podocytes

Podocyte proliferation was measured using the EdU assay kit (C0071S, Beyotime, Haimen, China). The podocytes were fixed with 0.5% buffered paraformaldehyde and then incubated with the EdU detection solution for 30 min in the dark. Hoechst (C0071S, Beyotime, Haimen, China) was applied to the podocytes, which were incubated in the dark for 10 min. Finally, cell proliferation was analyzed by fluorescence microscopy (Zeiss LSM 880 with airyscan, Zeiss, Berlin, Germany).

## 3. Results

### 3.1. WDR73 KO Inhibits the Proliferation of Cells

**Figure 1:**
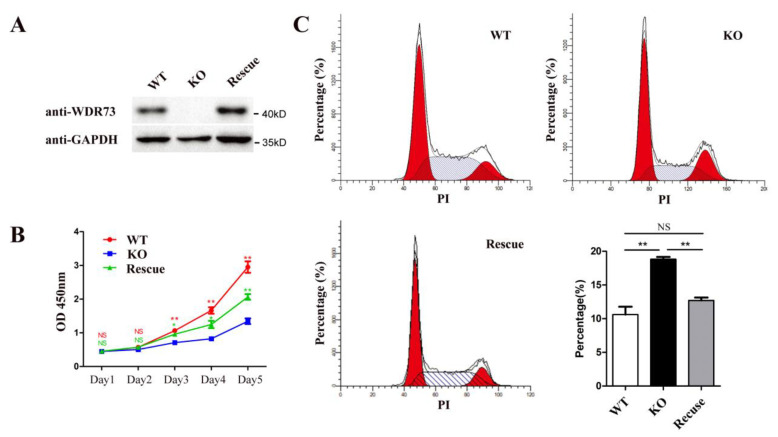
WDR73 KO cells are arrested in G2/M phase which can be rescued by WDR73 knock-in. (**A**) Expression of WDR73 in WT, WDR73 KO, and rescued cells; (**B**) growth curve of WT, WDR73 KO, and rescued cells; (**C**) cell cycle of WT, WDR73 KO, and rescued cells. The percentage of G2/M was calculated (** *p* < 0.01 (*t*-test), *n* = 4, error bars represent mean ± SEM). NS: not significant.

To investigate the role of WDR73 in the maintenance of normal cellular physiological functions, we knocked out WDR73 in HEK293 cells using CRISPR/Cas9 with a single-guide RNA (sgRNA) that targets exon 6 of WDR73 (Figure S1). Subsequently, knock-in with WT WDR73 was performed to further confirm the specific effect caused by WDR73 KO and eliminate off-target effects or other factors in the treatment process (Figure 1A). The effect of WDR73 on the growth and proliferation of cells was then investigated, and the results of the CCK-8 assay revealed that WDR73 KO cells showed a relatively slow growth rate compared with WT cells, which could be partly rescued by WDR73 knock-in (Figure 1B). Additionally, flow cytometry analysis indicated that WDR73 KO led to a remarkable increase in G2/M cell percentages. This blocking of G2/M could also be rescued by WDR73 knock-in (Figure 1C). However, apoptosis of KO cells was not significantly different from that of WT cells (Figure S2). These data indicated that WDR73 is essential for cell growth and proliferation.

### 3.2. WDR73 Plays a Critical Role in Cell Adhesion, Spreading, and Establishing Polarity Axis in Cell Division

**Figure 2:**
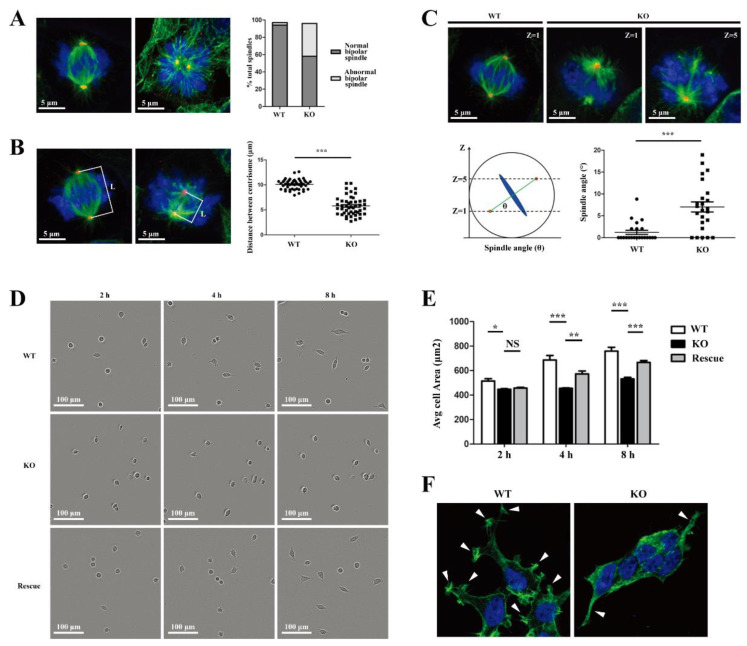
WDR73 KO causes uncontrolled spindle orientation and inhibits cell adhesion and spreading in cells. (**A**) WDR73 KO led to abnormal bipolar spindle formation. Immunofluorescence of mitotic spindles in WT and WDR73 KO cells with α-tubulin (green), γ-tubulin (red), and DNA (blue), Scale bar, 5 μm. The percentage of abnormal bipolar spindles was calculated. *n* > 100 cells from three independent experiments. (**B**) Spindle length was reduced in WDR73 KO cells. Left: maximum projections of WT and WDR73 KO cells stained for α-tubulin (green), γ-tubulin (red), and DNA (blue), L: distance between centrosome.Right: interpolar distances measured on single-plane confocal images. *n* > 100 cells from three independent experiments. *** *p* < 0.001 (*t*-test). Error bars represent mean ± SEM. (**C**) Uncontrolled spindle orientation in WDR73 KO cells. Left: immunofluorescence of the mitotic spindles in WT and WDR73 KO cells with α-tubulin (green), γ-tubulin (red), and DNA (blue). Representative images of z sections (0.299 um per stack) with maximum centrosome intensity in metaphase cells are shown. Scale bar: 5 μm. Middle: diagram of mitotic spindle angle calculated by 1/tan (the distance between centrosomes along the z axis/the distance between centrosomes in the xy plane). Right: The spindle angle was significantly increased in WDR73 KO cells (WT, *n* = 22; WDR73 KO, *n* = 24, *** *p* < 0.001 (*t*-test); error bars represent mean ± SEM). (**D**,**E**) Delayed adhesion and spreading in WDR73 KO cells. WT, WDR73 KO, and rescued cells were plated and monitored by IncuCyte. The images and average cell areas at 2, 4, and 8 h points are shown. *n* > 100 cells from three independent experiments. * *p* < 0.05, ** *p* < 0.01 and *** *p* < 0.001 (*t*-test). Error bars represent mean ± SEM. Scale bar: 100 μm. (**F**) Pseudopodia decreased in WDR73 KO cells. At 48 h after cell plating, microfilament was labeled with 488-phalloidin. White arrow: pseudopodia. Experiments were repeated three times. Scale bar: 20 μm. NS: not significant.

Due to the increase in the G2/M phase in WDR73 KO cells, we tried to define the potential roles of WDR73 in cell division and the features underlying delayed mitotic progression. Spindle morphology was examined by immunofluorescence with α-tubulin and γ-tubulin, revealing that the percentage of abnormal bipolar spindles of WDR73 KO cells significantly increased (Figure 2A and Figure S3). Some WDR73 KO cells exhibited shorter spindles than the WT cells (Figure 2B). Metaphase spindles in cultured adherent cells commonly adopt a planar orientation parallel to the surface of the culture dish. We observed that WT cells assembled a bipolar spindle oriented parallel to the substratum; in contrast, WDR73 KO cells had a bipolar spindle oriented unparallel to the substratum, such that spindle poles were detected in a different confocal z-axis. Our measurements of the spindle angle from planar orientation showed that WDR73 KO cells exhibited significantly larger spindle angles than WT cells (Figure 2C). These results indicate that WDR73 plays an important role in establishing spindle orientation and polarity axis for cell division.

Moreover, we observed that WDR73 KO caused changes in cell shape and size and inhibited cell adhesion and spreading on the dishes. Four hours after plating, a portion of WT and rescued cells adhered to the bottom of the plate, whereas the WDR73 KO cells maintained a round shape, floating in the medium. Another four hours later, most of the WT and rescued cells completed the adhesion process. Some of them even started cell division. Simultaneously, only a few parts of the KO cells began to adhere. WDR73 KO cells also exhibited delayed cell spreading and thus had a smaller projected area than WT cells (Figure 2D,E). At 48 h after cell plating, microfilaments were labeled with 488-phalloidin, showing that WDR73 KO cells displayed different cell morphologies and a lower degree of cell spreading compared to WT cells. Pseudopodia formation in WDR73 KO cells was significantly reduced by disturbed F-actin assembly (Figure 2F and Figure S4). These results suggested that WDR73 KO causes delayed cell adhesion and spreading, implying a role of WDR73 in cell polarization and migration.

### 3.3. The DEGs of WDR73 KO Cells Are Enriched in FA and Extracellular Matrix Pathway

**Figure 3:**
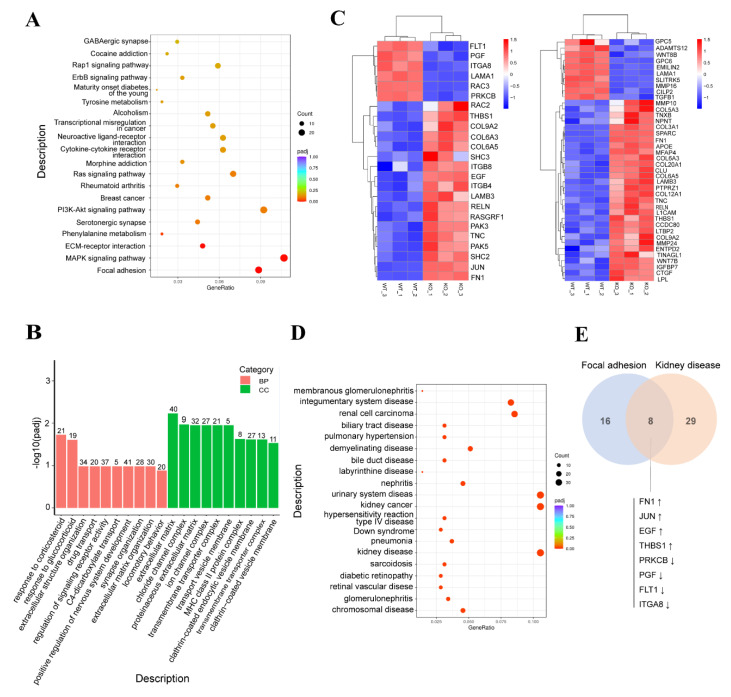
RNA sequencing analysis revealed differentially expressed genes (DEGs) enriched in two pathways. (**A**) KEGG pathway analysis of DEGs in WDR73 KO transcriptome. The top 20 most significantly enriched KEGG pathways are shown. (**B**) Gene Ontology (GO) functional clustering of DEGs. The top 10 most significantly affected categories are shown. BP, biological process; CC, cellular component). (**C**) The expression heatmap of focal adhesion and extracellular matrix-related genes. (**D**) Disease Ontology (DO) analysis of DEGs in WDR73 KO transcriptome. The top 20 most significantly enriched DO pathways are shown. (**E**) The Venn diagram used to identify the overlap DEGs of focal adhesion pathway and kidney disease, up arrow: up-regulated genes, down arrow: down-regulated genes. The ‘Count’ legend in (**A**,**D**) denotes the number of hit DEGs in the corresponding KEGG pathway or DO term.

RNA sequencing (RNA-seq) was used to provide insights into the transcriptome of the WDR73 KO cells. Three WDR73 KO samples and three WT samples were analyzed. The classical Bayesian algorithm was applied to identify DEGs, and volcano plots were used to visualize the variation in DEGs between WDR73 KO cells and WT cells. In total, 1042 mRNAs were differentially expressed in WDR73 KO cells, including 707 upregulated and 335 downregulated mRNAs (Figure S5). Gene enrichment analysis was used to assess the functional associations of DEGs derived from WDR73 KO cells. KEGG analysis results showed that DEGs were mostly enriched in FA, the MAPK signaling pathway, and ECM–receptor interaction (Figure 3A). Additionally, GO enrichment analysis results showed that the DEGs were mostly enriched in the extracellular matrix (ECM, belonging to cellular component) (Figure 3B). These analyses suggested that many of the DEGs were crucial for FA and ECM (Figure 3C). The two pathways had nine overlapping DEGs, which were all validated to be consistent with the RNA-seq data using qRT-PCR (Figure S6). Since FA complexes provide the main sites of cell adhesion to the ECM and are associated with the actin cytoskeleton, the interaction between FA and ECM plays a critical role in cell spreading, adhesion, and pseudopodia formation [11,12]. RNA-seq analysis revealed that the DEGs related to cell adhesion and ECM disturbed pseudopodia formation, which is supported by our previous findings that WDR73 KO inhibited cell adhesion and spreading.

Disease Ontology (DO) analysis results showed that DEGs could be significantly enriched in kidney disease (Figure 3D). This pathway and FA had significantly overlapping DEGs (*p* = 6.10 × 10^−7^) (Figure 3E), indicating that DEGs related to the FA pathway may be involved in kidney disease.

### 3.4. WDR73 Directly Interacts with PIP4K2C and Affects the PIP2 Generation

**Figure 4:**
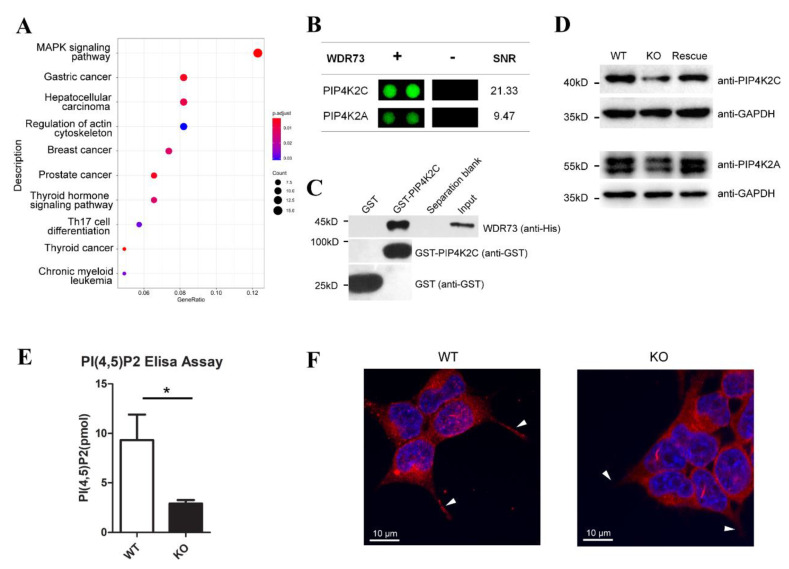
Identification of WDR73-binding proteins. (**A**) Pathway enrichment analysis by KEGG. Scatter plot of top 10 enriched KEGG pathways. (**B**) PIP4K2C and PIP4K2A signals from the protein arrays. Signal-to-noise ratios (SNRs) are shown (- = BSA control). (**C**) WDR73 interacts directly with PIP4K2C in vitro using GST pulldown assays. (**D**) WDR73 KO led to a significant reduction in the PIP4K2C and PIP4K2A proteins. (**E**) WDR73 KO disrupts the generation of PIP2. Cellular PIP2 levels from extracted acidic lipids were measured using the PIP2 mass ELISA kit. *n* = 3, * *p* < 0.05 (*t*-test). Error bars represent mean ± SEM. (**F**) WDR73 KO led to abnormal focal adhesion formation. Representative fluorescence images showing DNA (blue) and paxillin (red), white arrow: pseudopodia.

Although both the cell morphological alteration and DEG pattern suggest that WDR73 KO may disrupt FA and ECM-related pathways, the regulatory mechanism needs to be explored. We used the HuProt (Žilina, Slovakia) human protein microarray to identify potential WDR73-interacting proteins. A protein microarray composed of 21,000 purified human proteins as N-terminal GST fusions was incubated with purified human WDR73 protein. In total, 336 proteins were identified as potential WDR73-interacting proteins. To gain insight into the functional roles of WDR73, KEGG pathway analysis was conducted on 336 proteins to reveal their unique biological significance and key pathways. Potential WDR73-interacting proteins can be enriched in the regulation of the actin cytoskeleton pathway (Figure 4A), which plays an important role in cell adhesion, spreading, division, cell polarity, and spindle orientation [13,14].

Of the proteins involved in the regulation of the actin cytoskeleton pathway (Table S1), PIP4K2C generated the greatest signal-to-noise ratio (SNR 21.33) by protein microarray (Figure 4B). PIP4K2C and PIP4K2A (SNR 9.47) are two kinases generating PIP2, and previous immunoprecipitation experiments showed that PIP4K2C could interact with active PIP4K2A in vitro and can heterodimerize with PIP4K2A [15,16]. PIP2 plays a key role in restructuring the actin cytoskeleton [17,18]. Hence, we confirmed the potential direct interaction of WDR73 with PIP4K2C using the GST pulldown assay. Bacterially expressed GST-tagged PIP4K2C, but not GST alone, pulling His-tagged WDR73 down (Figure 4C) indicated that these two proteins can interact directly with each other. Moreover, Western blotting showed that WDR73 KO led to a significant reduction in the PIP4K2C and PIP4K2A protein levels, which can be rescued partly by WDR73 knock-in (Figure 4D). Thus, we assessed whether the decrease in PIP4K2C and PIP4K2A levels caused by WDR73 KO actually reduced intracellular PIP2. As shown in Figure 4E, WDR73 KO significantly reduced intracellular PIP2 production. Evidence suggests that PIP2 plays important roles in regulating actin reorganization and FA assembly [18,19,20]. To investigate the formation of FAs in cells, cells were stained with an anti-paxillin antibody, which showed that FAs were widely distributed and aggregated into spots in WT cells. However, the intensity of paxillin foci decreased remarkably in the pseudopodia of WDR73 KO cells, which might indicate a decrease in FA formation (Figure 4F and Figure S7). The decreased FA was not due to the reduced expression of FA core proteins such as talin, paxillin, vinculin, and FAK (Figure S8). As several kinases, including type I (PIP5K) and type II (PIP4K) kinase isoforms (A, B, and C), are involved in generation of PIP2, we also investigated the mRNA transcription level of these kinases in different organs of the mice. Results revealed the highest transcription of PIP4K2C among the tested kinases in the kidney (Figure S9), implying that it has a specific physiological function in the kidney and may participate in the progression of kidney disease in GAMOS1.

### 3.5. WDR73 Can Interact with EPG5 and Control PIP4K2C Degradation by Autophagy

**Figure 5:**
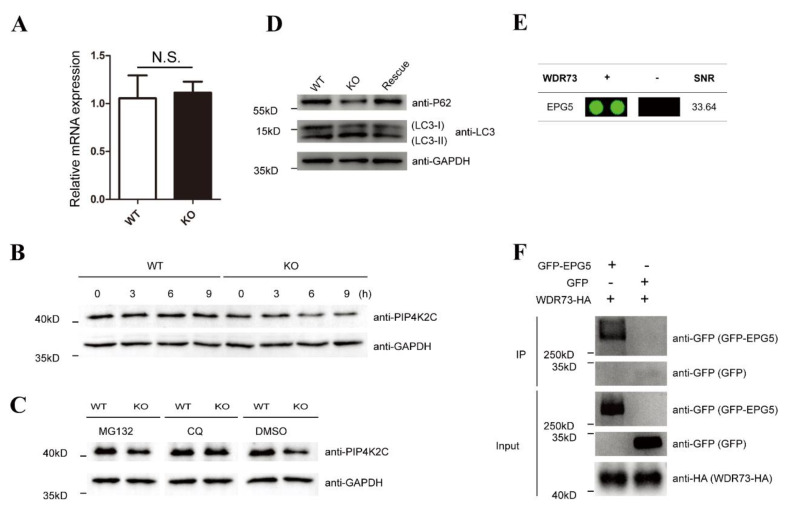
Interaction between WDR73 and EPG5, and autophagy-dependent degradation of PIP4K2C. (**A**) PIP4K2C was not regulated by the WDR73 at the transcriptional level. Total mRNA from WDR73 KO and control cells was extracted and quantitative real-time (qRT–PCR) assays were performed. *n* = 3. Error bars represent the mean ± SEM. (**B**) The half-life of PIP4K2C decreased in WDR73 KO cells by cycloheximide chase assay. Cells were treated with CHX (100ug/mL) for 0, 3, 6, and 9 h before harvest, followed by Western blot analysis. (**C**) WDR73 KO promoted autophagy–lysosomal degradation of PIP4K2C. Cells were treated with DMSO, MG132 (20 μM), or chloroquine (50 μM) for 12 h. (**D**) WDR73 KO enhanced autophagy flux. Western blot analysis of LC3-I/II and p62 levels. (**E**) A direct interaction between WDR73 and EPG5 was shown based on HuProt human protein microarray. (**F**) Co-IP assay showed an interaction between WDR73 and EPG5. N.S.: not significant.

Since there was a significant reduction in PIP4K2C and PIP4K2A protein levels due to the WDR73 depletion, the mechanism underlying the decrease in such proteins induced by WDR73 KO was systematically explored. First, quantitative real-time PCR analysis showed that WDR73 depletion did not significantly affect PIP4K2C mRNA levels (Figure 5A); therefore, downregulation of PIP4K2C by WDR73 KO should occur at the post-transcriptional stage. Subsequently, we assessed the PIP4K2C stability in WDR73 KO cells after treatment with cycloheximide (Figure 5B), a protein synthesis inhibitor. The results showed that the PIP4K2C protein degraded faster in WDR73 KO cells, suggesting that the stability of PIP4K2C was impaired by WDR73 depletion. The ubiquitin–proteasome system (UPS) and autophagy–lysosomal pathway (ALP) are the two major protein degradation systems in eukaryotic cells [21]. When WDR73 KO and WT cells were treated with the UPS inhibitor MG132 or the ALP inhibitor chloroquine, downregulation of PIP4K2C by WDR73 depletion was restored by chloroquine, but not by MG132 (Figure 5C), indicating that WDR73 might affect the autophagy–lysosome-dependent degradation of PIP4K2C. To determine if the WDR73 KO would induce an abnormal increase in autophagy, the abundance of microtubule-associated protein 1 light chain 3 II (LC3II) and autophagy substrate protein p62 was detected. Western blotting showed that the rate of LC3-I to LC3-II conversion increased, and p62 levels were dramatically reduced in WDR73 KO cells (Figure 5D). By re-querying the data from the previous human proteome microarray, a protein named ectopic P-granules autophagy protein 5 (EPG5) with a high SNR (SNR: 33.64) was also identified to potentially interact with WDR73 (Figure 5E). The Co-IP assay also confirmed the interaction between WDR73 and EPG5 (Figure 5F). These results revealed that WDR73 KO could enhance autophagic flux and reduce the stability of PIP4P2C and PIP4K2A proteins through EPG5.

### 3.6. Podocyte-Specific WDR73 Depletion Mice Are more Susceptible to Glomerular Injury

**Figure 6:**
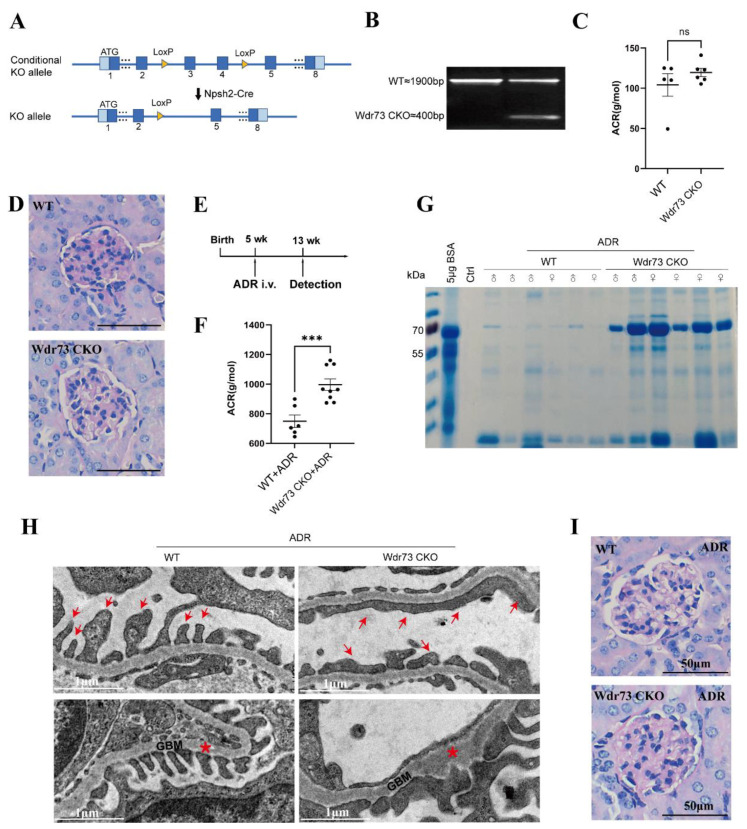
WDR73 was required for podocyte FP maintenance. (**A**) Schematic depicted generation of conditional knockout mice in which WDR73 is specifically ablated in podocytes by crossing Wdr73flox/flox with Nphs2Cre mice. (**B**) PCR genotyping of genomic DNA from the isolated glomeruli of Wdr73 CKO mice. Wild-type (upper band) and exon 3 and exon 4 deletion (lower band) alleles of the Wdr73 gene. (**C**,**D**) Loss of WDR73 did not result in an obvious renal phenotype, assessed by measurement of albuminuria and histologic analysis (PAS stain) up to an age of 20 weeks (each dot indicates an individual animals. ACR, albumin–creatinine ratio. Scale bars, 50 μm each. (**E**) Schematic depicting the timeline of the ADR nephropathy model in Wdr73 CKO mice. (**F**,**G**) Quantitation of urinary ACR and SDS–PAGE analysis showed increased levels of proteinuria in ADR-treated WT and Wdr73 CKO mice at 13 weeks (dots indicate individual animals), *** *p* < 0.001 (*t*-test). (**H**) Representative TEM images of glomeruli in ADR-treated WT and Wdr73 CKO mice at 13 weeks. Compared with WT mice, Wdr73 CKO mice exhibited increasing GBM (red asterisks) and pronounced widening and effacement of podocyte FPs (red arrows). Scale bars, 1 μm each. (**I**) PAS-stained kidney sections of ADR-treated WT and Wdr73 CKO mice at 13 weeks. Scale bars, 50 μm each. All TEM and histological (PAS) data were verified in at least 3 WT mice and 3 Wdr73 CKO mice. NS: not significant.

To further confirm the role of Wdr73 in vivo, we first generated WDR73 systemic KO mice. However, no Wdr73-/- embryos and pups were detected at any given period (Figure S10), suggesting that loss of WDR73 is embryonically lethal in mice, and WDR73 might play an important role in early development. Podocytes are essential for the maintenance of the glomerular filtration barrier. Therefore, we generated a podocyte-specific conditional knockout mouse of Wdr73 (Wdr73 CKO) by crossing Wdr73flox/flox with Nphs2-Cre to study the role of WDR73 in podocytes (Figure 6A,B). The Wdr73 CKO mice did not show any growth or histologic lesion abnormalities, and they exhibited no increase in proteinuria levels up to the age of 20 weeks (Figure 6C,D and Figure S11A,B). Then, we applied Adriamycin (ADR) to induce glomerular injury both for the WT and Wdr73 CKO strains to confirm the role of WDR73 in maintaining podocyte function (Figure 6E). We found that loss of WDR73 significantly increased the susceptibility of podocytes toward injury by ADR, which was characterized by the detection of high levels of albuminuria and podocyte injury in Wdr73 CKO mice at 5 weeks after ADR injection. ADR-treated Wdr73 CKO mice exhibited increased albuminuria levels in both male and female Wdr73 CKO mice compared with the same treated WT mice of the same sex (Figure 6F,G). Podocyte injury was evidenced by glomerular basement membrane (GBM) thickening and podocyte foot process (FP) broadening and effacement by transmission electron microscopy (TEM) analysis (Figure 6H). However, kidneys of ADR-treated Wdr73 CKO mice did not show obvious histologic alterations in the glomerular or tubular compartment (Figure 6I and Figure S11C). Together, these observations suggested that WDR73 deficiency causes impaired podocyte FP formation, resulting in an impaired filtration barrier in the kidney.

### 3.7. WDR73 Depletion Influences Focal Adhesion Assembly in Podocytes

**Figure 7:**
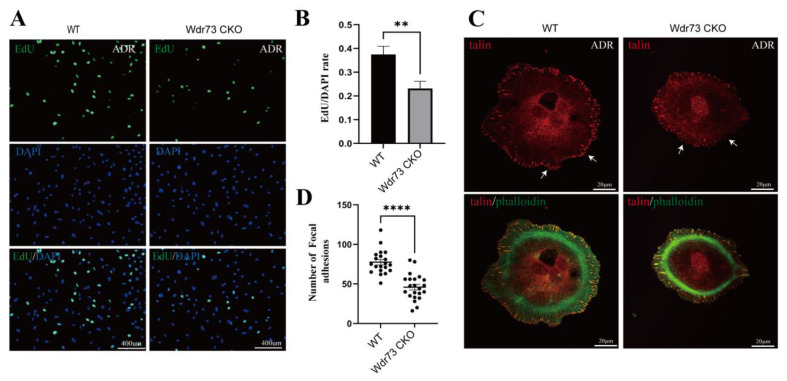
WDR73 was required for focal adhesion assembly in podocytes. (**A**,**B**) WDR73 depletion inhibited podocyte proliferation. Proliferating ADR-treated mice primary podocytes were labeled with EdU (green) and cell nuclei were stained with DAPI (blue). *n* = 3, ** *p* < 0.01 (*t*-test). Error bars represent mean ± SEM. Scale bars, 400 μm each. (**C**,**D**) WDR73 KO led to abnormal focal adhesion formation. Disrupted focal adhesions (white arrows) and quantification of the number of FAs per cell are indicated in ADR-treated mice primary podocytes. Focal adhesion marker talin (red), microfilament (green), *n* = 20, **** *p* < 0.0001 (*t*-test). Error bars represent mean ± SEM. Scale bars, 20 μm each.

To further dissect the effect of WDR73 deletion in podocytes, we established a primary podocyte culture system, and cells were confirmed to be positive for nephrin expression before the assay (Figure S12). First, we labeled the cells with the proliferation marker EdU, and the results showed that the proliferation rate of primary podocytes isolated from ADR-treated Wdr73 CKO mice was reduced compared with WT cells (Figure 7A,B). In addition, ADR-treated Wdr73 CKO mice primary podocytes showed marked differences in FAs when compared to WT podocytes. FAs were widely distributed and assembled into spots in the WT podocytes, whereas in the Wdr73 CKO mouse primary podocytes, the FA structure was disrupted, and the number and size of speckle-like adhesion spots were reduced (Figure 7C,D).

## 4. Discussion

In this study, we revealed that WDR73 exerts an essential function in the regulation of focal adhesion and the actin cytoskeleton and provides a novel molecular mechanism in reduced renal cell proliferation, adhesion, and spreading. By human protein microarray assay and GST pulldown, we confirmed that WDR73 can directly interact with PIP4K2C, which usually heterodimerizes with PIP4K2A and catalyzes the phosphorylation of phosphatidylinositol 5-phosphate (PI5P) to generate PIP2. WDR73 depletion led to a significant reduction in the PIP4K2C and PIP4K2A proteins, consequently inducing a dramatic decrease in intracellular PIP2.

PIP2 plays a key role in FA assembly and actin polymerization [18]. FA complexes provide the main sites for cell adhesion to the ECM and are associated with the actin cytoskeleton, which is required for cell adhesion and spindle orientation in cell division [14,22,23]. Actually, FA and ECM alterations in podocytes have been widely reported to be one of the main cellular bases for proteinuria. FA is a key signal and structural hub of foot processes that regulate the actin cytoskeleton, through which podocytes could firmly bind to the GBM, which is a dense matrix of ECM components. Once the FA dynamic or assembly is disrupted, podocytes gradually detach from the GBM, leading to the irreversible progression of kidney disease [24,25,26]. We demonstrated that there were significant changes in cell morphology of WDR73 KO cells, with significantly reduced cell spreading area and less pseudopod formation. The adhesion ability also decreased dramatically. We observed that the number of speckle-like adhesion spots was significantly reduced and the shape was ambiguous in WDR73 KO cells. Considering the RNA-seq results, the enrichment of DEGs in FA and ECM-related pathways together, these results suggest that FA and actin filament assembly were disrupted by the absence of WDR73, leading to pseudopod formation, cell adhesion, and spreading abnormality, which may be the primary cellular pathological basis for the albuminuria and other renal abnormalities in GAMOS1. Although this point has not been mentioned in other studies on GAMOS, we confirmed that the FA formation and distribution were impaired in primary podocytes derived from ADR-injected Wdr73 CKO mice. In fact, despite the resistant C57BL/6 background, Wdr73 CKO animals with only a single dose of ADR manifested increased albuminuria levels, GBM thickening, and podocyte effacement, all suggesting podocyte injury. Taken together, these findings strongly indicate that WDR73 plays key physiologic and cell biologic roles in focal adhesion.

PIP4K2C is ubiquitously expressed at different levels in tissues, with primary expression in the kidney, brain, heart, and testes [15]. However, the role of PIP4K2C in kidney development and disease remains unclear. PIP4K2C can heterodimerize with PIP4K2A and has been suggested to serve as a chaperone for the more active isoforms, and its presence likely affects the distribution and enzyme activity of the 2A and 2B isoforms [16,27]. We confirmed that WDR73 can directly interact with PIP4K2C, and we found that the mRNA expression of PIP4K2C was higher in the mouse kidney than in other organs. WDR73 KO led to a significant reduction in the PIP4K2C and PIP4K2A proteins, resulting in the reduction in intracellular PIP2 and disruption of FA and actin filament assembly. These results may provide new clues regarding the role of interaction between PIP4K2C and WDR73 in the kidney.

Considering the reduction in PIP4K2C induced by WDR73 depletion, the stability of PIP4K2C was assessed. Our results revealed the unstable PIP4K2C should be eliminated mainly through enhanced autophagy, but not the ubiquitin–proteasome system. An autophagy protein EPG5 was subsequently proved to also interact with WDR73. EPG5 is a Rab7 effector that determines the fusion specificity of autophagosomes with late endosomes/lysosomes and is a key autophagy regulatory protein [28]. We intended to further confirm these molecular interactions with WDR73 in vivo using mice for further verification.

## 5. Conclusions

In summary, our study suggested that WDR73 can interact with PIP4K2C to form a complex with a function in FA assembly and regulates PIP4K2C protein stability through the autophagy–lysosomal pathway. This study would provide a new perspective on the molecular mechanism of WDR73 in primary albuminuria and other kidney abnormalities of GAMOS1.

## Data Availability

All data supporting the reported result in this study can be found in the Supplementary Materials.

## Supplementary Materials

The following supporting information can be downloaded at: https://www.mdpi.com/article/10.3390/biology11101397/s1, Figure S1: Sanger sequencing trace of the WDR73 sgRNA target site; Figure S2: Apoptosis of KO cells was not significantly different from that of WT cells. (A) Flow cytometric dot plots of WT and WDR73 KO cells. (B) The apoptotic percentage was normal in WT and WDR73 KO cells. N = 3; Figure S3: WDR73 KO led to abnormal bipolar spindle formation. Immunofluorescence of mitotic spindles in WT and WDR73 KO cells with α-tubulin (green), γ-tubulin (red), and DNA (blue). White arrow: normal bipolar spindle; yellow arrow: abnormal bipolar spindle; Figure S4: The number of pseudopodia was calculated. N = 150 cells from three independent experiments, ** *p* < 0.01; Figure S5: A volcano plot illustrating DEGs from RNA-seq analysis between the WT and WDR73 KO cells. Genes upregulated and downregulated are shown in red and green. Values are presented as the log2 of tag counts; Figure S6: qRT-PCR validation analysis shows the mRNA expression of focal adhesion and ECM-associated genes. n = 3, ** *p* < 0.01 (t-test), error bars represent mean ± SEM; Figure S7: The number of focal adhesions was calculated. N = 60 cells from three independent experiments, ** *p* < 0.01; Figure S8: The decreased FA was not due to the reduced expression of FA-related proteins. Expression of talin, FAK, paxillin, and vinculin in WT and WDR73 KO cells; Figure S9: qRT-PCR on tissues of WT mice of adult stage. The mRNA expression of PIP4K2C was highest in the adult mice kidney. Expression levels of PIP4K2A, PIP4K2B, PIP4K2C, PIP5K2A, PIP5K2B, and PIP5K2C were normalized for β-actin. N = 3; Figure S10: WDR73 knockout mice died at early embryonic day. (A) Schematic representation of wild-type (WT) and frameshift mutation alleles. Arrows below the scheme represent primer sets for WT (PII and PIII) and frameshift mutation (PI and PIV) alleles used in B. (B) Representative result of PCR genotyping. PCR genotyping of genomic DNA from the mice was performed in two sets of primers to detect wild-type (lower band) and frameshift mutation (upper band) alleles of the wdr73 gene. (C) Genotyping results of wdr73 mutant embryos and pups; Figure S11: (A) Growth curves of WT (n = 8) and Wdr73 CKO (n = 5) mice. (B) Representative renal histologic analysis (Masson stain) of WT (n = 8) and Wdr73 CKO (n = 8) mice at 20 weeks. (C) Representative renal histologic analysis (Masson stain) of WT (n = 5) and Wdr73 CKO (n = 5) mice at 13 weeks, sacrificed 5 weeks after ADR injection (20 mg/kg, i.v.); Figure S12: (A) Schematic of primary podocyte culture system. (B) Isolated glomeruli mice primary podocytes were labeled with nephrin; Figure S13: full western bolt figures of Figure 1A; Figure S14: full western bolt figures of Figure 4C; Figure S15: full western bolt figures of up panel in Figure 4D; Figure S16: full western bolt figures of down panel in Figure 4D; Figure S17: full western bolt figures of Figure 5B; Figure S18: full western bolt figures of Figure 5C; Figure S19: full western bolt figures of Figure 5D; Figure S20: full western bolt figures of Figure 5F; Figure S21: full western bolt figures of Figure S. Table S1: The proteins involved in the regulation of the actin cytoskeleton pathway. SNR, signal-to-noise ratio; Table S2: Primers used for qRT-PCR analysis of the mRNA expression of focal adhesion and ECM-associated genes; Table S3: Primers used for qRT-PCR analysis of the mRNA expression of PIP4K2A, PIP4K2B, PIP4K2C, PIP5K2A, PIP5K2B, and PIP5K2C in mouse tissues; Table S4: Antibodies used in this study; Table S5: Primers used for mouse experiments.
