# Supplementary material for: WDR73 Depletion Destabilizes PIP4K2C Activity and Impairs Focal Adhesion Formation in Galloway–Mowat Syndrome
Source: Biology (Basel). 2022 Sep 25;11(10):1397. doi: 10.3390/biology11101397 (PMC9598763; doi:10.3390/biology11101397)
Supplement: Supplementary file 1 [file biology-11-01397-s001.zip › biology-1929926-supplementary.pdf]

WDR73 Depletion Destabilizes PIP4K2C Activity and Impairs Focal Adhesion Formation in Galloway–Mowat Syndrome

SUPPLEMENTARY FIGURES

Figure S1

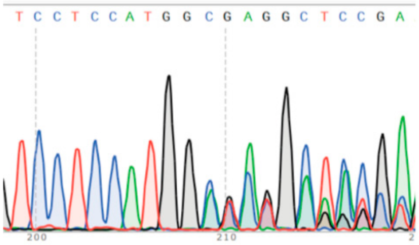

Figure S1: Sanger sequencing trace of the WDR73 sgRNA target site.

Figure S2

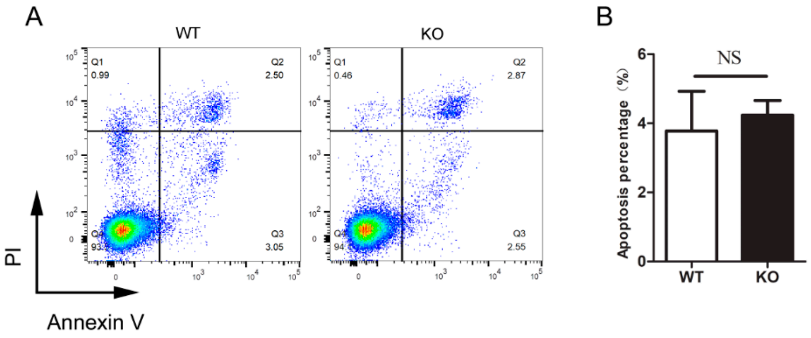

Figure S2: Apoptosis of KO cells was not significantly different from that of WT cells. (A) Flow cytometric dot plots of WT and WDR73 KO cells. (B) The apoptotic percentage was normal in WT and WDR73 KO cells. *n*=3.

Figure S3

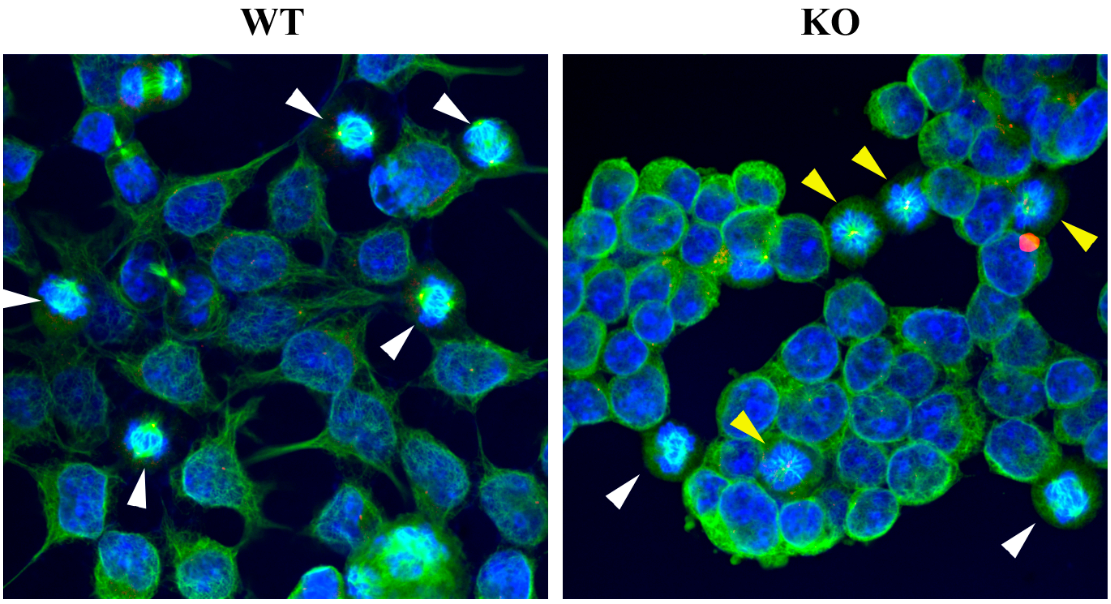

Figure S3: WDR73 KO led to abnormal bipolar spindle formation. Immunofluorescence of mitotic spindles in WT and WDR73 KO cells with α-tubulin (green), γ-tubulin (red), and DNA (blue). White arrow: normal bipolar spindle; yellow arrow: abnormal bipolar spindle.

Figure S4

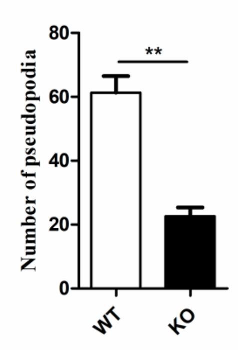

Figure S4: The number of pseudopodia was calculated. n=150 cells from three independent experiments, $^{**}p < 0.01$.

Figure S5

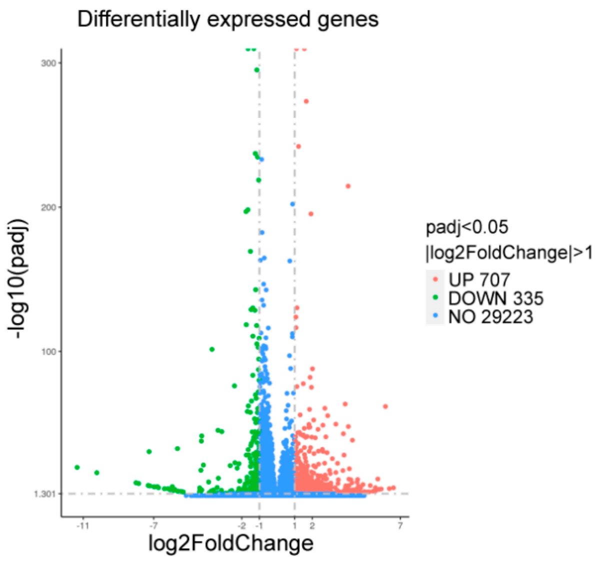

Figure S5: A volcano plot illustrating DEGs from RNA-seq analysis between the WT and WDR73 KO cells. Genes upregulated and downregulated are shown in red and green. Values are presented as the log2 of tag counts.

Figure S6

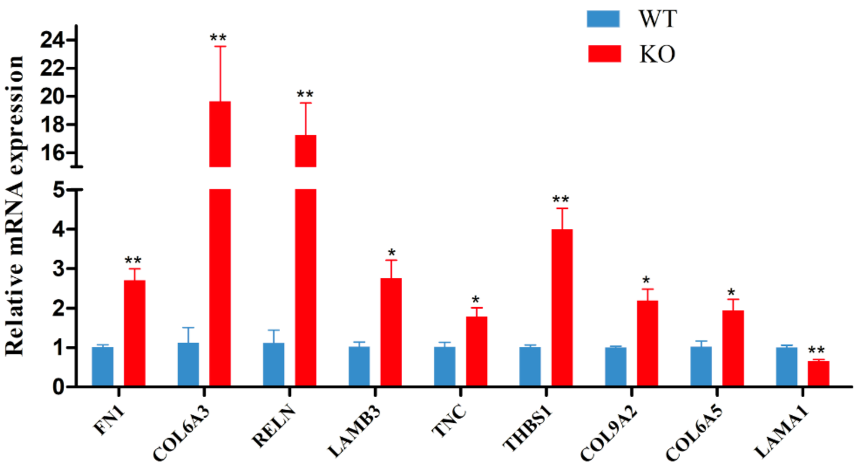

Figure S6 qRT-PCR validation analysis shows the mRNA expression of focal adhesion and ECM-associated genes. $n = 3$, $^{**}p < 0.01$ (t-test), error bars represent mean±SEM.

Figure S7

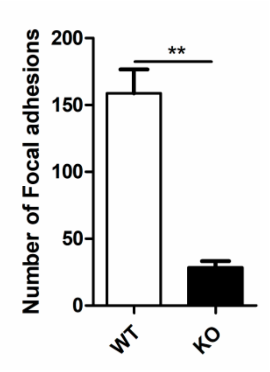

Figure S7: The number of focal adhesions was calculated. n=60 cells from three independent experiments, **p < 0.01.

Figure S8

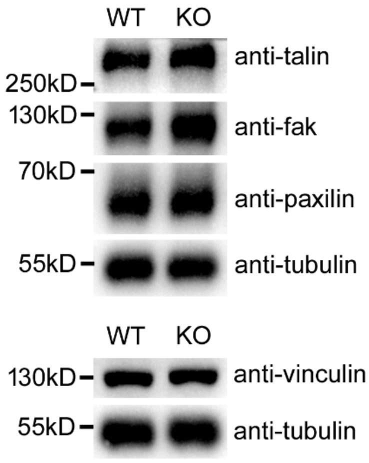

Figure S8: The decreased FA was not due to the reduced expression of FA-related proteins. Expression of talin, FAK, paxillin, and vinculin in WT and WDR73 KO cells.

Figure S9

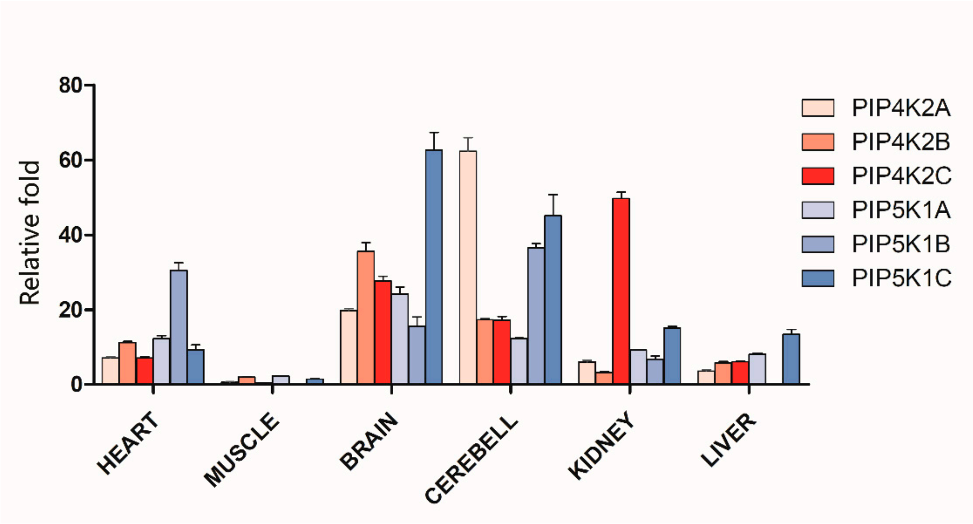

Figure S9: qRT-PCR on tissues of WT mice of adult stage. The mRNA expression of PIP4K2C was highest in the adult mice kidney. Expression levels of PIP4K2A, PIP4K2B, PIP4K2C, PIP5K2A, PIP5K2B, and PIP5K2C were normalized for β-actin. *n*=3.

Figure S10

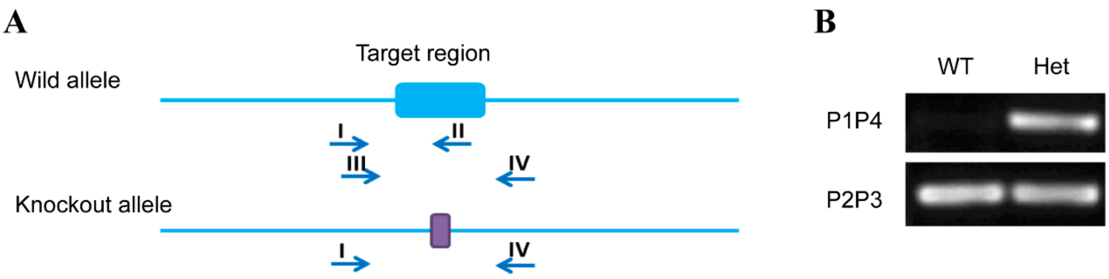

C

Genotyping of mutant wdr73 embryos and pups			
Age	wdr73+/+	wdr73+/-	wdr73-/-
P7	34	71	0
E14.5	3	8	0
E10.5	13	42	0
E:Embryonic day P:Postnatal mice			

Figure S10: WDR73 knockout mice died at early embryonic day. (A) Schematic

representation of wild-type (WT) and frameshift mutation alleles. Arrows below the scheme represent primer sets for WT (PII and PIII) and frameshift mutation (PI and PIV) alleles used in B. (B) Representative result of PCR genotyping. PCR genotyping of genomic DNA from the mice was performed in two sets of primers to detect wild-type (lower band) and frameshift mutation (upper band) alleles of the *wdr73* gene. (C) Genotyping results of *wdr73* mutant embryos and pups.

Figure S11

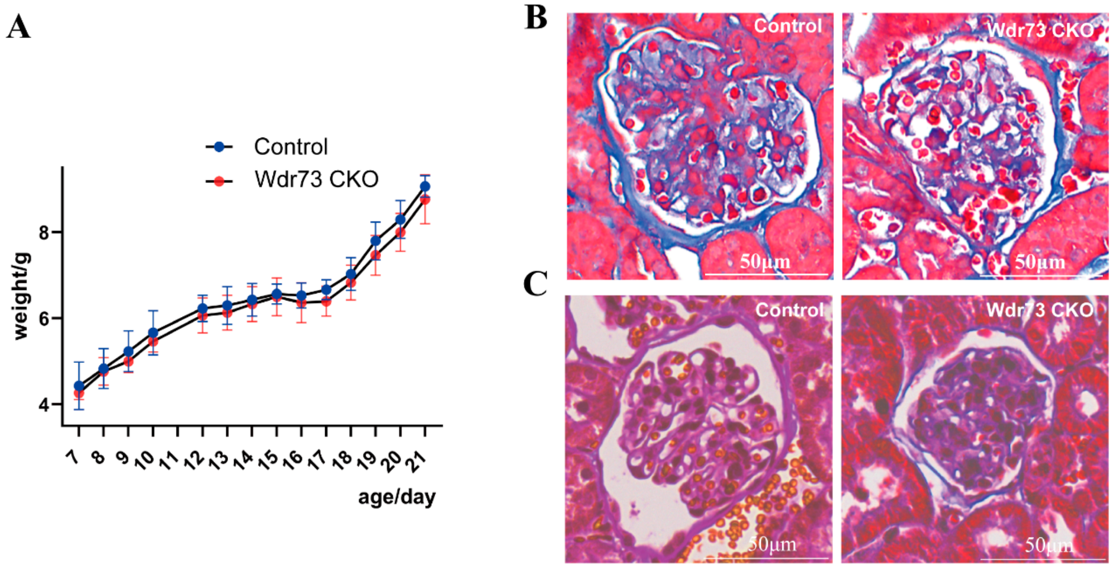

Figure S11

(A) Growth curves of WT (n=8) and Wdr73 CKO (n = 5) mice. (B) Representative renal histologic analysis (Masson stain) of WT (n = 8) and Wdr73 CKO (n = 8) mice at 20 weeks. (C) Representative renal histologic analysis (Masson stain) of WT (n = 5) and Wdr73 CKO (n = 5) mice at 13 weeks, sacrificed 5 weeks after ADR injection (20 mg/kg, i.v.).

Figure S12

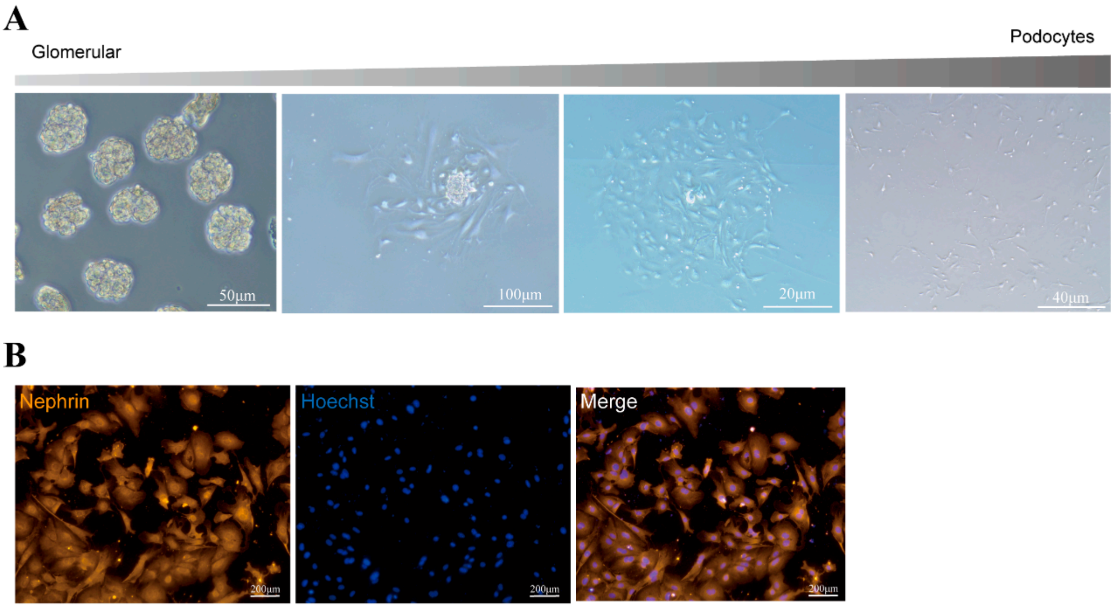

Figure S12

(A) Schematic of primary podocyte culture system. (B) Isolated glomeruli mice primary podocytes were labeled with nephrin.

Original images for gels and blots which have been cropped or used in the main text and supplementary materials are presented

Figure S13

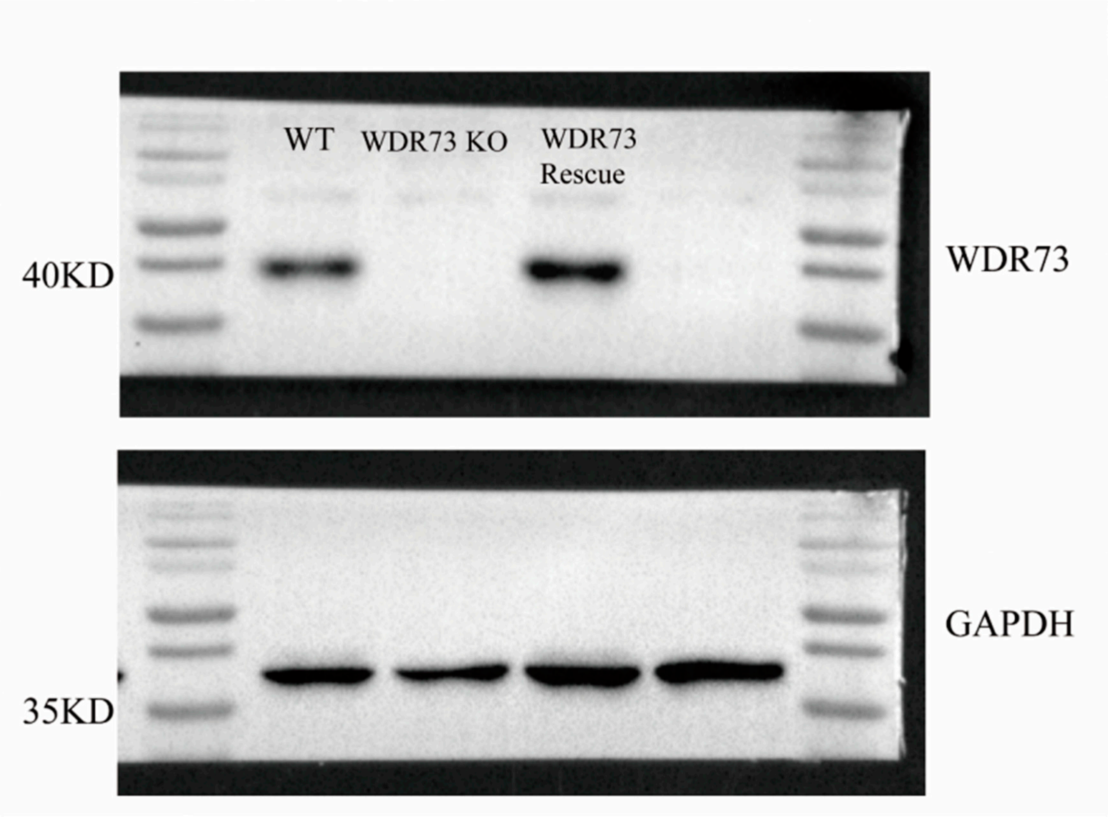

Figure S13: Full western bolt figures of Figure 1A

Figure S14

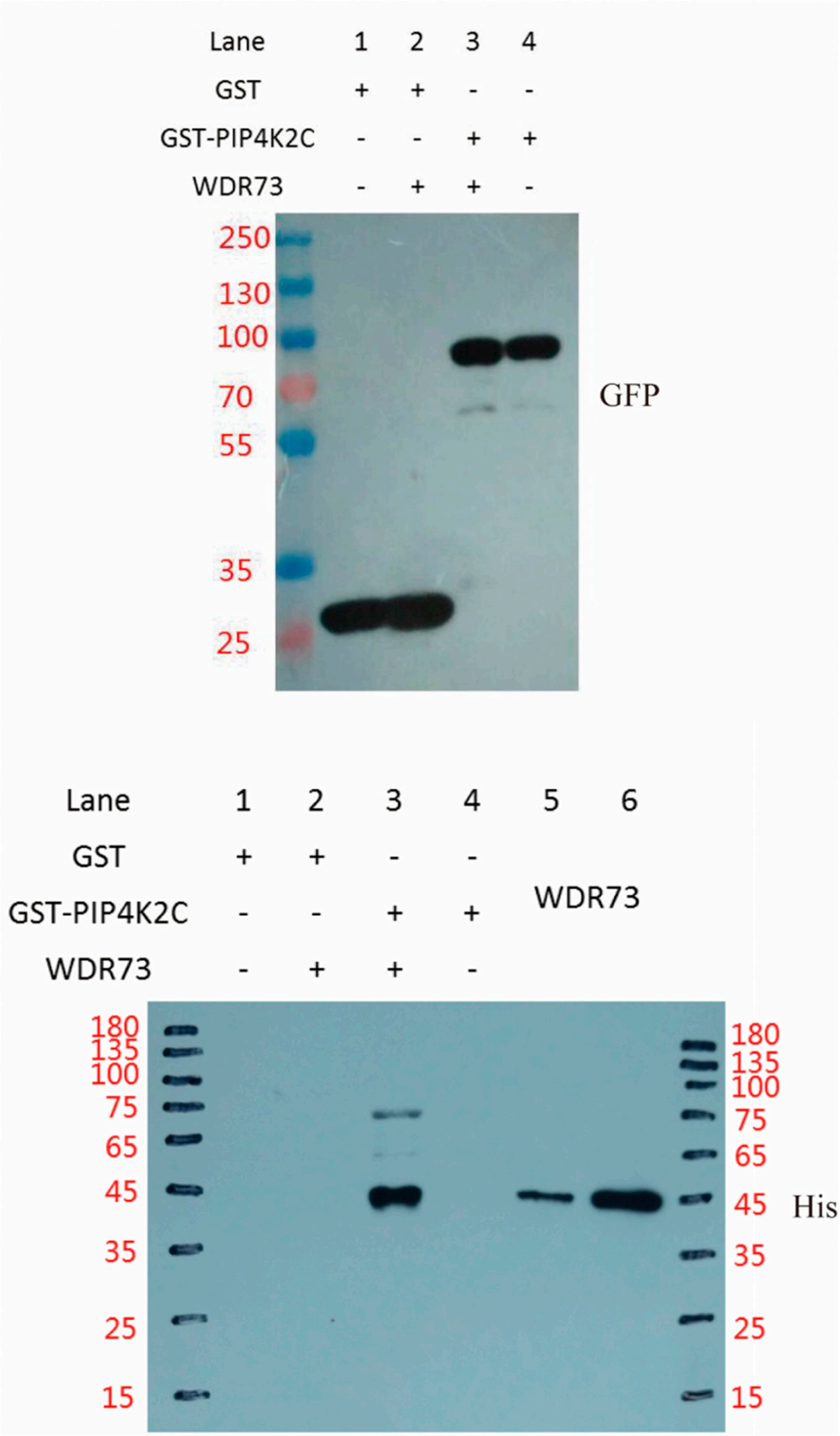

Figure S14: Full western bolt figures of Figure 4C

Figure S15

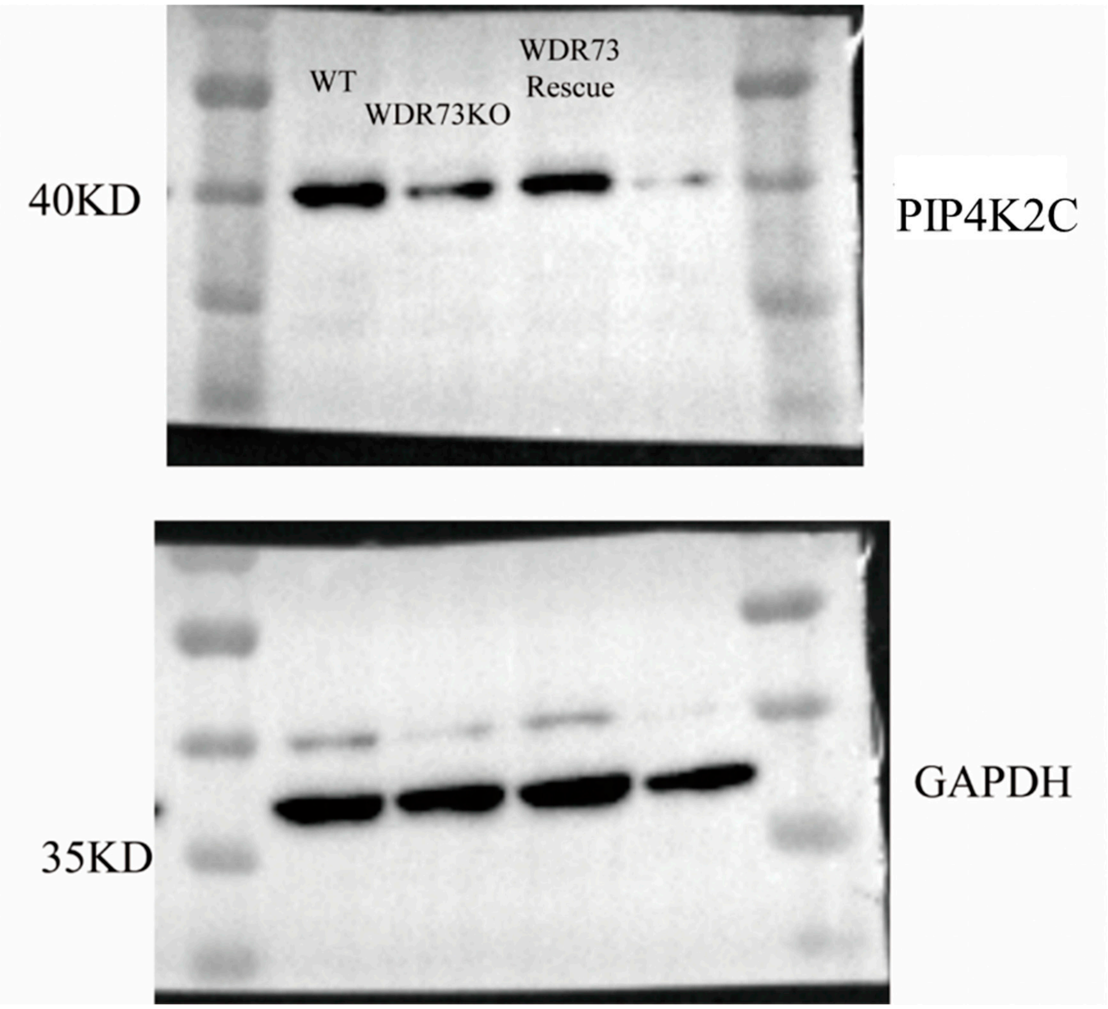

Figure S15: Full western bolt figures of up panel in Figure 4D

Figure S16

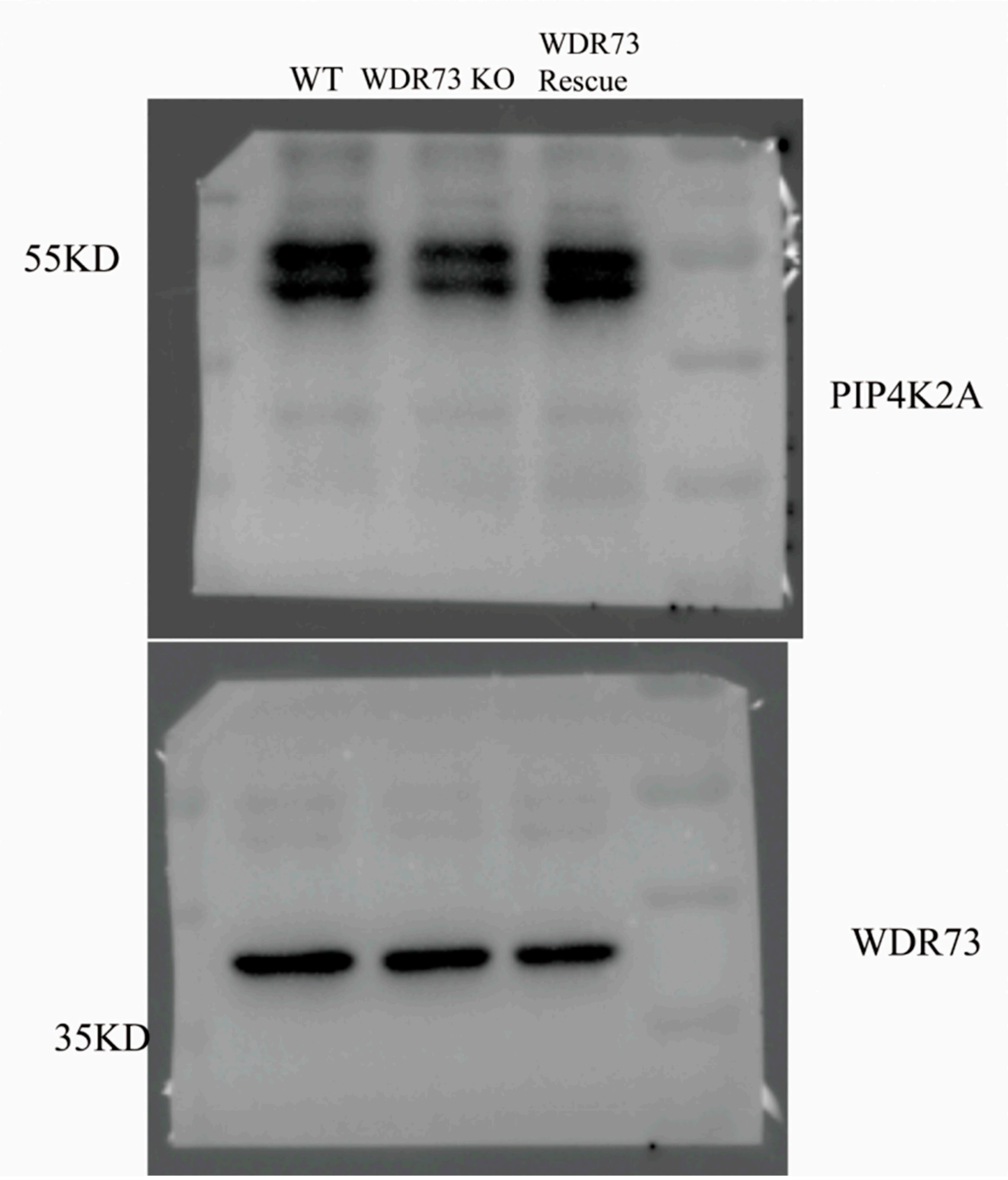

Figure S16: Full western bolt figures of down panel in Figure 4D

Figure S17

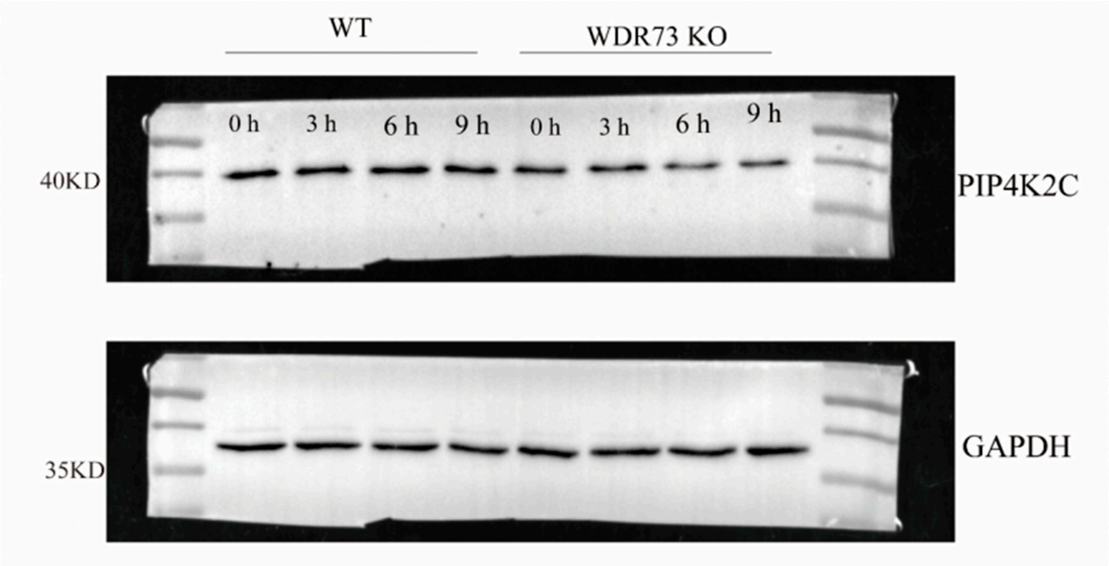

Figure S17: Full western bolt figures of Figure 5B

Figure S18

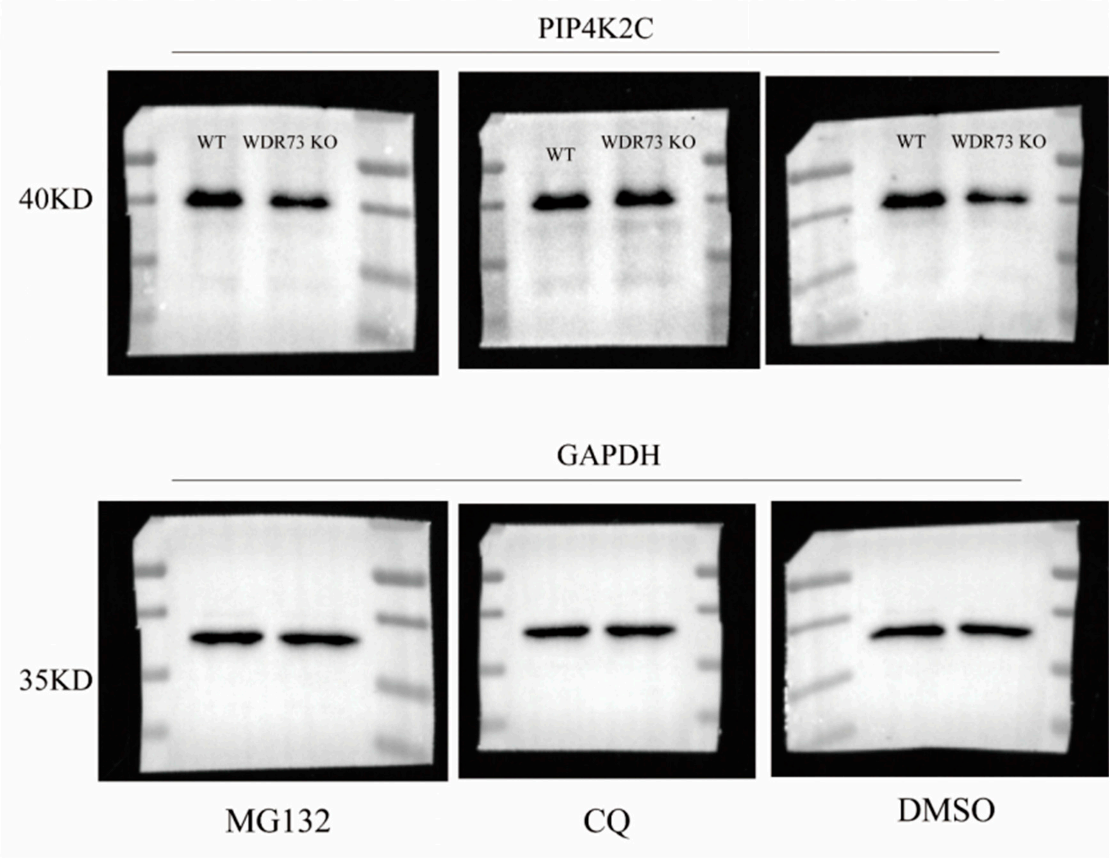

Figure S18: Full western bolt figures of Figure 5C

Figure S19

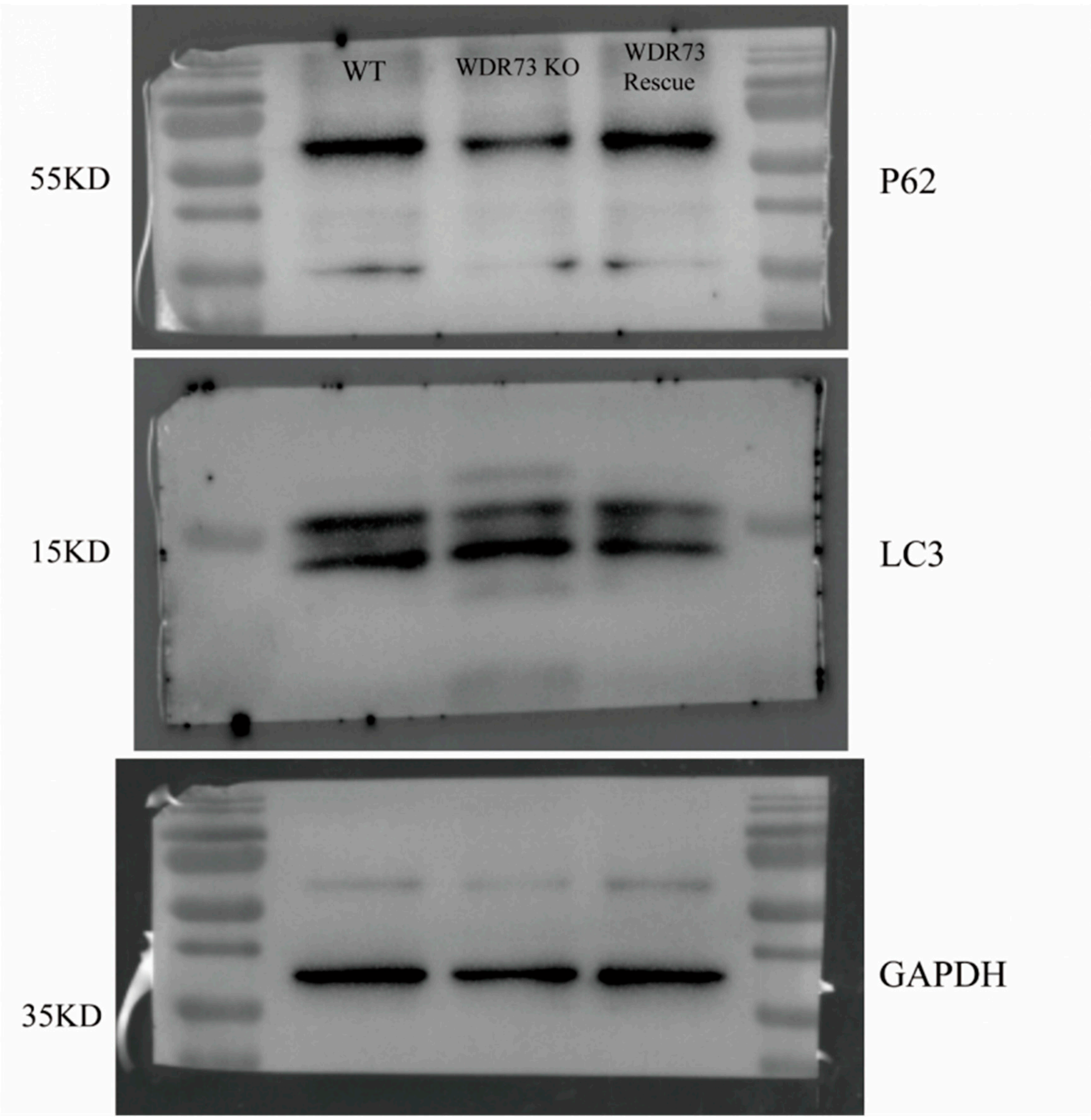

Figure S19: Full western bolt figures of Figure 5D

Figure S20

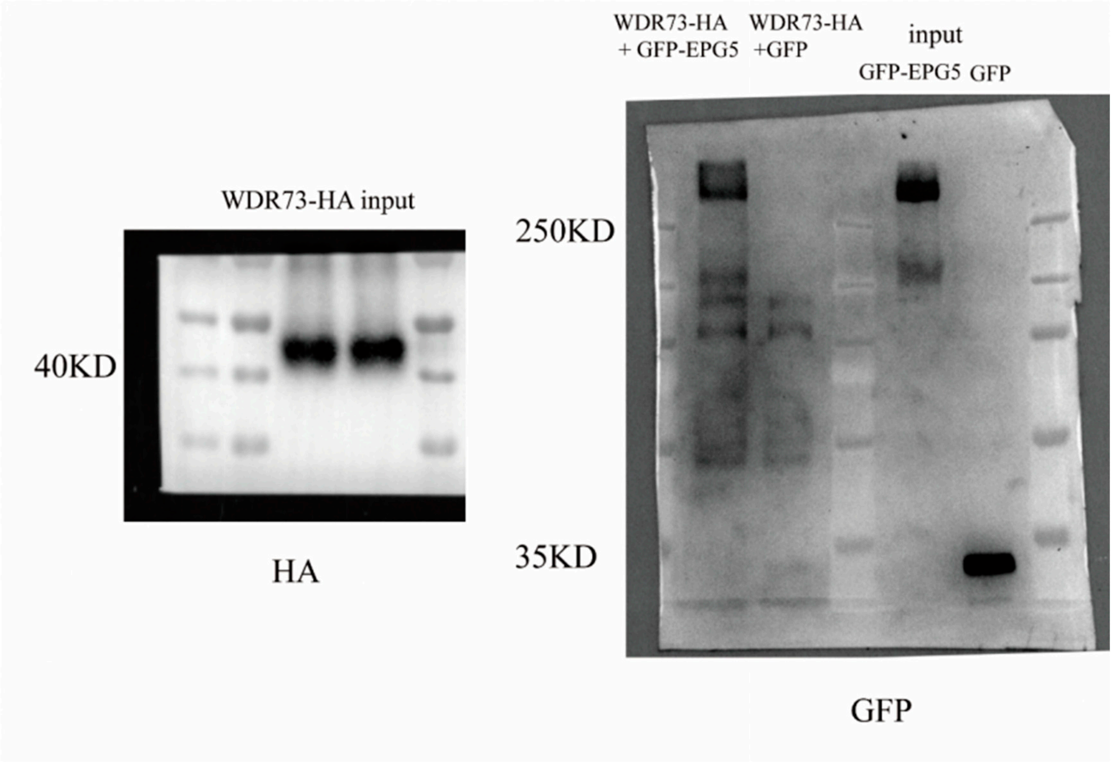

Figure S20: Full western bolt figures of Figure 5F

Figure S21

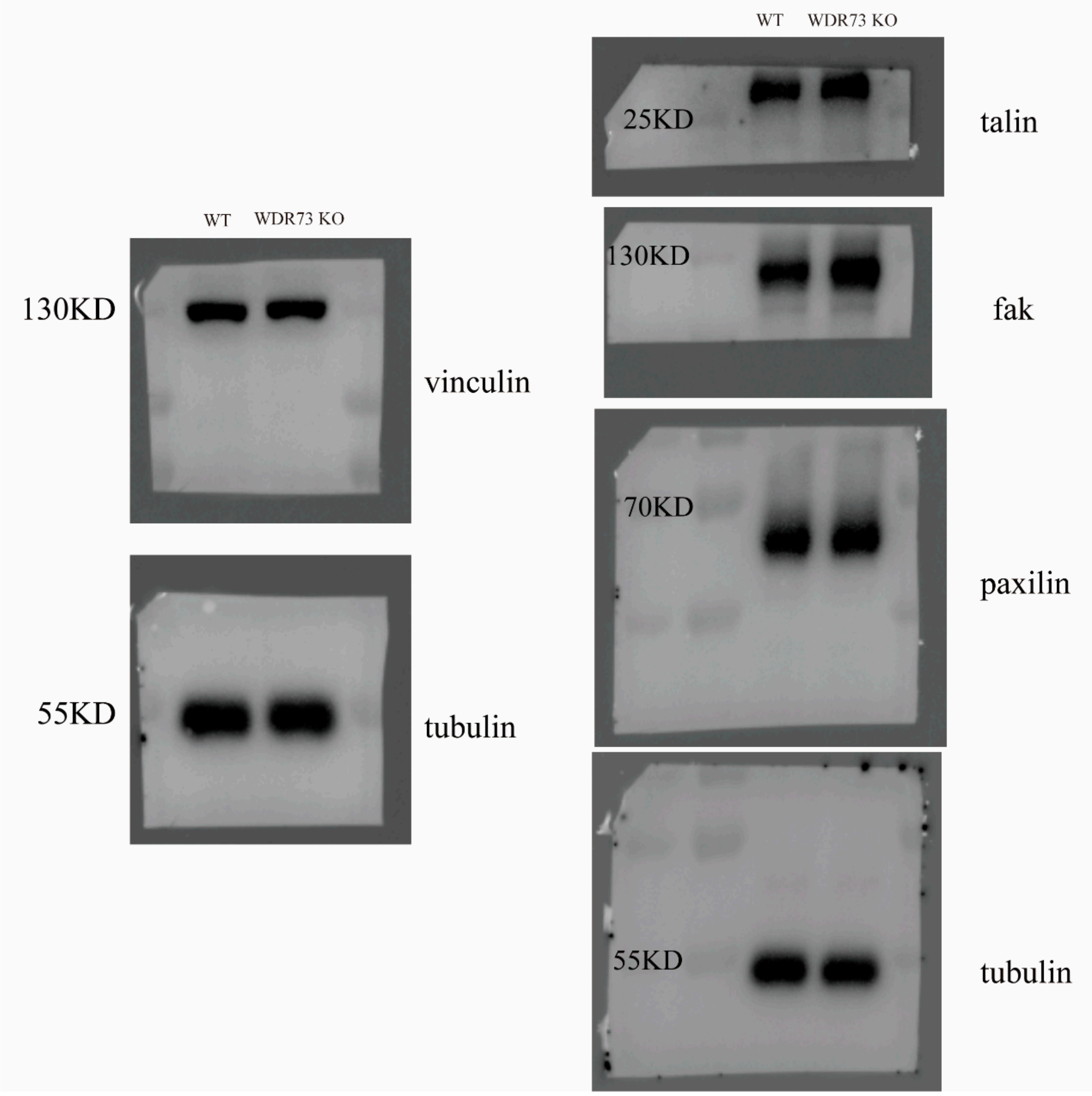

Figure S21: Full western bolt figures of Figure S8

SUPPLEMENTARY TABLES

Table S1

Gene name	SNR
PIP4K2C	21.33
FGF9	11.54
SSH3	11.03
PIP4K2A	9.47
EGFR	8.00
CRK	7.91
RRAS	6.88

CRKL	6.55
F2	6.12
MSN	6.04

Table S1: The proteins involved in the regulation of the actin cytoskeleton pathway. SNR, signal-to-noise ratio.

Table S2

Primer name	Sequence (5′-3′)
COL6A3-QPCR-F	CATAACCGCTGTGCGGAAAAT
COL6A3-QPCR-R	TCATCTAGGGACTTACCACCTG
LAMB3-QPCR-F	GCAGCCTCACAACTACTACAG
LAMB3-QPCR-R	CCAGGTCTTACCGAAGTCTGA
FN1-QPCR-F1	CGGTGGCTGTCAGTCAAAG
FN1-QPCR-R1	AAACCTCGGCTTCCTCCATAA
TNC-QPCR-F	TCCCAGTGTTCGGTGGATCT
TNC-QPCR-R	TTGATGCGATGTGTGAAGACA
JUN-QPCR-F2	AACAGGTGGCACAGCTTAAAC
JUN-QPCR-R2	CAACTGCTGCGTTAGCATGAG
THBS1-QPCR-F	AGACTCCGCATCGCAAAGG
THBS1-QPCR-R	TCACCACGTTGTTGTCAAGGG
COL9A2-QPCR-F	TCCCTGGTGAGATTGGAATCC
COL9A2-QPCR-R	TTGGTTGGACACAGGAAATCC
ITGB4-QPCR-F	CTCCACCGAGTCAGCCTTC
ITGB4-QPCR-R	CGGGTAGTCCTGTGTCCTGTA
ITGB8-QPCR-F	ACCAGGAGAAGTGTCTATCCAG
ITGB8-QPCR-R	CCAAGACGAAAGTCACGGGA
LAMA1-QPCR-F	GTGATGGCAACAGCGCAAA
LAMA1-QPCR-R	GACCCAGTGATATTCTCTCCCA
RELN- QPCR-F	ACATCTACAAGTGTTCAGGCATC
RELN- QPCR-R	TGGTTACCAAACTGGTGGTCA
COL6A5- QPCR-F	TGCGCTGAACCTTCGACTG
COL6A6- QPCR-R	AGCGTGGAAATTGTCTGTTCTG
GAPDH- QPCR-F	GTGAAGGTCGGAGTCAACGGA
GAPDH- QPCR-R	GGCAACAATATCCACTTTACCAGAG

Table S2: Primers used for qRT-PCR analysis of the mRNA expression of focal adhesion and ECM-associated genes.

Table S3

Primer name	Sequence (5′-3′)
PIP4K2A-QPCR-F	AAGAGTCTGATGCCAAGAACCTGT
PIP4K2A-QPCR-R	TGCAGTGCAACTTAAGGATGGTAA
PIP4K2B-QPCR-F	CATCCTCACAGAAGAACATGGC
PIP4K2B-QPCR-R	CCTGGTCATTCACCGTCTCA
PIP4K2C-QPCR-F	CATCTTCCACTGCTAATGTGTCTCC
PIP4K2C-QPCR-R	TTGAGTTATGGCTCTGACTCCTCTCT
PIP5K1A-QPCR-F	ACCTGAAGGGTTCAACTTACAAG
PIP5K1A-QPCR-R	ACAGTCACGCTGTAGAGTCTT
PIP5K1B-QPCR-F	TGCCAAAATTCTATGGGCTGT
PIP5K1B-QPCR-R	GGATGCTCTTCGCTTGTATGT
PIP5K1C-QPCR-F	ATTTCCGCTTCAAGACCTATGC
PIP5K1C-QPCR-R	GGTGACGTAGAAGACAGAGCC
β-actin-QPCR-F	CCTGTGCTGCTCACCGAGGC
β-actin-QPCR-R	TGGCTGGGGTGTTGAAGGTCTC

Table S3: Primers used for qRT-PCR analysis of the mRNA expression of PIP4K2A, PIP4K2B, PIP4K2C, PIP5K2A, PIP5K2B, and PIP5K2C in mouse tissues.

Table S4

Antibody name	Company
anti-gamma tubulin (ab11316)	Abcam
anti-talin 1 (ab157808)	Abcam
anti-paxillin (ab32084)	Abcam
anti-WDR73 (HPA039357)	Sigma-Aldrich
anti-PIP5K2C (WH007983M1)	Sigma-Aldrich
anti-vinculin(V9131)	Sigma-Aldrich
anti-FLAG® M2 (A9469)	Sigma-Aldrich
anti-Nephrin (ABT331)	Sigma-Aldrich
anti-His-Tag(66005-1-Ig)	Proteintech

anti-GFP tag (66002-1-Ig)	Proteintech
anti-LC3 (14600-1-AP)	Proteintech
anti-P62, SQSTM1 (18420-1-AP)	Proteintech
anti-GST (2624)	Cell Signaling Technology
anti-PIP4K2A(5527S)	Cell Signaling Technology
anti-HA (3725)	Cell Signaling Technology
anti-FAK(71433T)	Cell Signaling Technology

Table S4: Antibodies used in this study.

Table S5

Primer name	Sequence (5′-3′)
guide RNA1	TGTGACATCTGCAGCGTAGCTGG
guide RNA2	GATCCTGTTGTGAAGCACCCGGG
PI	AGGAGACGGAGACAGGAGGATAGA
PII	GAGGAGGAGGTCAAAGCACAGGTC
PIII	CCTGACCATCTGGCAATCTAAT
PIV	TCCTGCAGCTGTGAACCTACCT
Wdr73-5F	TCTGTGTCTTCCTCTAGGAAGGTG
Wdr73-5R	TCTGATGGCCTCTTCTTCTGG
Wdr73-3F	CTTGAACTTCCTCCAGGAGTTG
Wdr73-3R	GACAGAATCAACACCTCCTGCTC
Nphs2-cre-F	CGGTTATTCAACTTGCACCA
Nphs2-cre-R	GCGCTGCTGCTCCAG
Gapdh-F	AGGTCGGTGTGAACGGATTTG
Gapdh-R	GGGGTCGTTGATGGCAACA
Wdr73-F	GGATTGGTGAAAAAGGAGTCTTTG
Wdr73-R	TCCTCCATGGCGTACTTTGA

Table S5: Primers used for mouse experiments.
